# Triterpenoid acids isolated from *Schinus terebinthifolia* fruits reduce *Staphylococcus aureus* virulence and abate dermonecrosis

**DOI:** 10.1038/s41598-020-65080-3

**Published:** 2020-05-15

**Authors:** Huaqiao Tang, Gina Porras, Morgan M. Brown, Francois Chassagne, James T. Lyles, John Bacsa, Alexander R. Horswill, Cassandra L. Quave

**Affiliations:** 1 Center for the Study of Human Health, Emory University College of Arts and Sciences, 30322 Atlanta, Georgia USA; 2 Department of Pharmacy, Sichuan Agricultural University, Chengdu, Sichuan China; 3 Department of Immunology and Microbiology, University of Colorado School of Medicine, Aurora, CO 80045 USA; 4 X-ray Crystallography Center, Emory University College of Arts and Sciences, Atlanta, GA 30322 USA; 5 Department of Dermatology, Emory University School of Medicine, Atlanta, 30322 GA USA

**Keywords:** Drug discovery, Chemical biology, Bacteriology

## Abstract

*Staphylococcus aureus* relies on quorum sensing to exert virulence to establish and maintain infection. Prior research demonstrated the potent quorum sensing inhibition effects of “430D-F5”, a refined extract derived from the fruits of *Schinus terebinthifolia*, a medicinal plant used for the traditional treatment of skin and soft tissue infections. We report the isolation and identification of three compounds from 430D-F5 that reduce virulence and abate dermonecrosis: 3-oxo-olean-12-en-28-oic acid (**1**), 3-oxotirucalla-7,24Z-dien-26-oic acid (**2**) and 3α-hydroxytirucalla-7,24 Z-dien-27-oic acid (**3**). Each compound inhibits all *S. aureus* accessory gene regulator (*agr*) alleles (IC_50_ 2–70 μM). Dose-dependent responses were also observed in *agr-*regulated reporters for leucocidin A (*lukA*, IC_50_ 0.4-25 μM) and glycerol ester hydrolase or lipase (*gehB*, IC_50_ 1.5–25 μM). Surprisingly, dose-dependent activity against the nuclease reporter (*nuc*), which is under the control of the *sae* two-component system, was also observed (IC_50_ 0.4–12.5 μM). Compounds **1-3** exhibited little to no effect on the *agr-*independent *mgrA P2* reporter (a constitutive promoter from the *mgrA* two-component system) and the esxA reporter (under control of *mgrA*). Compounds **1**-**3** inhibited δ-toxin production *in* vitro and reduced dermonecrosis in a murine *in vivo* model. This is the first report of triterpenoid acids with potent anti-virulence effects against *S. aureus*.

## Introduction

*Staphylococcus aureus* has long been recognized as a significant cause of both community-acquired (CA) and healthcare associated (HA) infections, such as endocarditis, septic arthritis, osteomyelitis, and necrotizing pneumonia^1^. In the past decade, an increasing number of infections by methicillin-resistant *S. aureus* (MRSA) have been documented and estimates in the United States suggest that MRSA causes between 11,000–18,000 deaths and 80,000 invasive infections annually^2,3^. Furthermore, a single clone of CA-MRSA (USA300) has emerged as the most common cause of all skin and soft tissue infections in the United States and continues to pose a serious public health threat in community and healthcare settings^4,5^. With the last novel class of antibiotics to be brought to market being discovered in the 1980s, new strategies are necessary to respond to the widespread development of antibiotic resistant infections^6^. Some promising future approaches include promoting infection prevention to reduce the need for antibiotics, encouraging investment in antimicrobial agents with new regulatory policies and economic models, slowing the spread of resistance in order to preserve the useful lives of available antibiotics, and developing novel therapeutics that modulate host-microbe interactions without placing direct selective pressures known to drive resistance resistance^7^.

*S. aureus* antibiotic resistance can be due to various extrinsic, or acquired, mechanisms such as enzymatic drug modification, mutated drug targets, enhanced efflux pump expression, and altered membrane permeability^8^. Additionally, intrinsic mechanisms of resistance such as biofilm formation and production of virulence factors that subvert the host immune response, play a key role in prolonging and increasing the pathogenicity of *S. aureus* infections. The production of a suite of superantigens, toxins and exo-enzymes heavily contribute to the invasive nature of staphylococcal infections, and this virulence mechanism is largely controlled by quorum sensing (QS).

*S. aureus* uses multiple two-component systems to sense and respond to changes in cell density and environmental cues. One of these two-component systems is the Accessory Gene Regulator (*agr*) system, which has been extensively characterized for its complex regulatory role in global MRSA virulence and its requirement for MRSA skin infection pathogenesis^1,2,9^. *Agr* senses and responds to its cognate auto-inducing peptide (AIP) signal in a cell-density dependent manner. All described AIP signals are between 7 and 12 amino acids long, with the C-terminal amino acids constrained in a thiolactone or lactone ring and an N-terminal “tail” extension^10,11^. *S. aureus* has four allelic variants of the *agr* system (*agr*-I, II, III, IV), and each recognizes and responds to its cognate AIP signal (AIP-I, II, III, IV). The dominant CA-MRSA USA300 is *agr* Type-I, while health-care associated MRSA strains are predominantly *agr* Type-II^12,13^.

The *agrBDCA* operon encodes the AIP precursor (AgrD) and integral membrane protease (AgrB) necessary for final AIP processing, as well as the membrane-localized histidine kinase sensor (AgrC) and response regulator (AgrA). A hypervariable region spanning *agrBDC* determines the *agr* type. At sufficient local concentration, cognate AIPs bind AgrC, which dimerizes and phosphorylates the response regulator AgrA. Downstream, activated AgrA binds chromosomal promoters P2 and P3 to induce the transcription of the *agrBDCA* operon and the small RNA regulator RNAIII, respectively. As the primary effector of the *agr* system, RNAIII post-transcriptionally regulates the expression of more than 200 virulence-associated genes including toxins (α, β, δ, and γ), proteases, lipases, superantigens (toxic shock syndrome toxin-1, enterotoxins B, C, and D), and leukocidins^10,14–16^. Additionally, the RNAIII transcript includes the *hld* gene for δ-toxin, an amphipathic 26 amino acid peptide with cytolytic activity.

Given such extensive *agr*-dependent virulence factor production, inhibition of *agr* signaling (i.e. quorum sensing) has been proposed as an alternative strategy to prevent or treat MRSA skin infections. Several promising molecules have been reported, including the AgrA inhibitor savarin^3^, AgrB inhibitor ambuic acid^4^, and the pan-*agr* inhibitor apicidin^5^. Previously, we reported on the quorum sensing inhibitory activity of a refined extract from the fruit of the Brazilian peppertree (*Schinus terebinthifolia* Raddi, Anacardiaceae) against several MRSA strains^6^. *S. terebinthifolia* is an evergreen shrub native to South and Central America and grows as a noxious weed in the southern United States. It was introduced to the USA just over 100 years ago as an ornamental plant, and has since spread via a process of stratified dispersal around established populations and by long-distance jumps due to human activities^17^. In Florida, where we collected it for the present study, *S. terebinthifolia* is listed as a Category I invasive exotic species throughout the state^18^. Eradication efforts are underway in Florida, and have included largescale removal in the Everglades, application of herbicides, and most recently, deployment of parasitizing insects as a biocontrol measure^19^.

However, in Brazil where the plant is valued as a medicinal species and popularly known as “pimenta-rosa”, it is used in folk medicine for the treatment of an array of illnesses, including several associated with infection and inflammation. Several parts of the plant have been found to contain chemicals with antimicrobial^20,21^, anti-inflammatory^22^, antioxidant^22,23^ and anti-tumor^24^ bioactivities. The current study characterizes the anti-virulence activity of three triterpenoid acids isolated from the fruit of *S. terebinthifolia* against clinically-relevant MRSA strains.

## Results

### Bioassay-guided isolation of three bioactive triterpenoid acids

Bioassay-guided fractionation of an organic extract of *S. terebinthifolia* fruits (named extract 430) was directed by a set of reporter strain assays of MRSA *agr*::P3 activation for *agr* types I-IV^25^. Inhibition of YFP fluorescence in these strains was used as a preliminary indication for the inhibition of quorum sensing. An aqueous suspension of 430 was sequentially partitioned against hexanes (430B), then ethyl acetate (430 C). The final remaining water layer (430 F) was determined to be the most bioactive under these testing parameters and was selected for further fractionation with a flash chromatography system using a gradient of hexane, ethyl acetate and methanol. The most active fraction, 430F-F5, was chosen for compound isolation via reverse phase HPLC using a gradient system of water and methanol. A first round of HPLC yielded 12 fractions (430F-F5-PF1 through PF12). The most bioactive fractions, 430F-F5-PF11 and 430F-F5-PF12, were selected for a second round of HPLC fractionation, which led to the isolation of three active triterpenoid acids. An overview of the fractionation strategy, with percent yields of the active fractions and compounds is provided (Supplementary Fig. 1).

**Figure 1:**
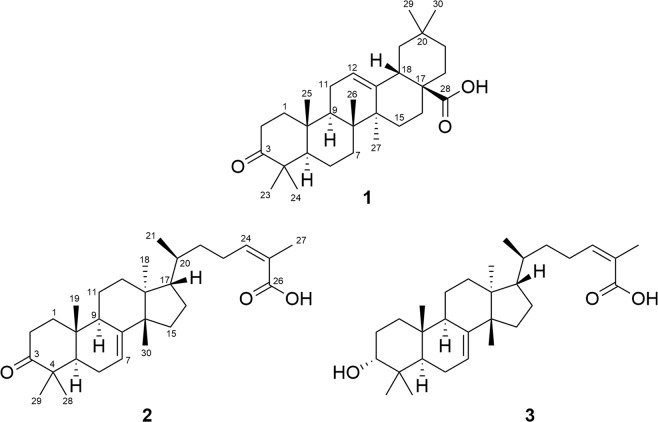
Structures of isolated bioactive compounds: 3-oxo-olean-12-en-28-oic acid (**1**), 3-oxotirucalla-7,24*Z*-dien-26-oic acid (**2**) and 3α-hydroxytirucalla-7,24*Z*-dien-26-oic acid (**3**).

**Figure 2:**
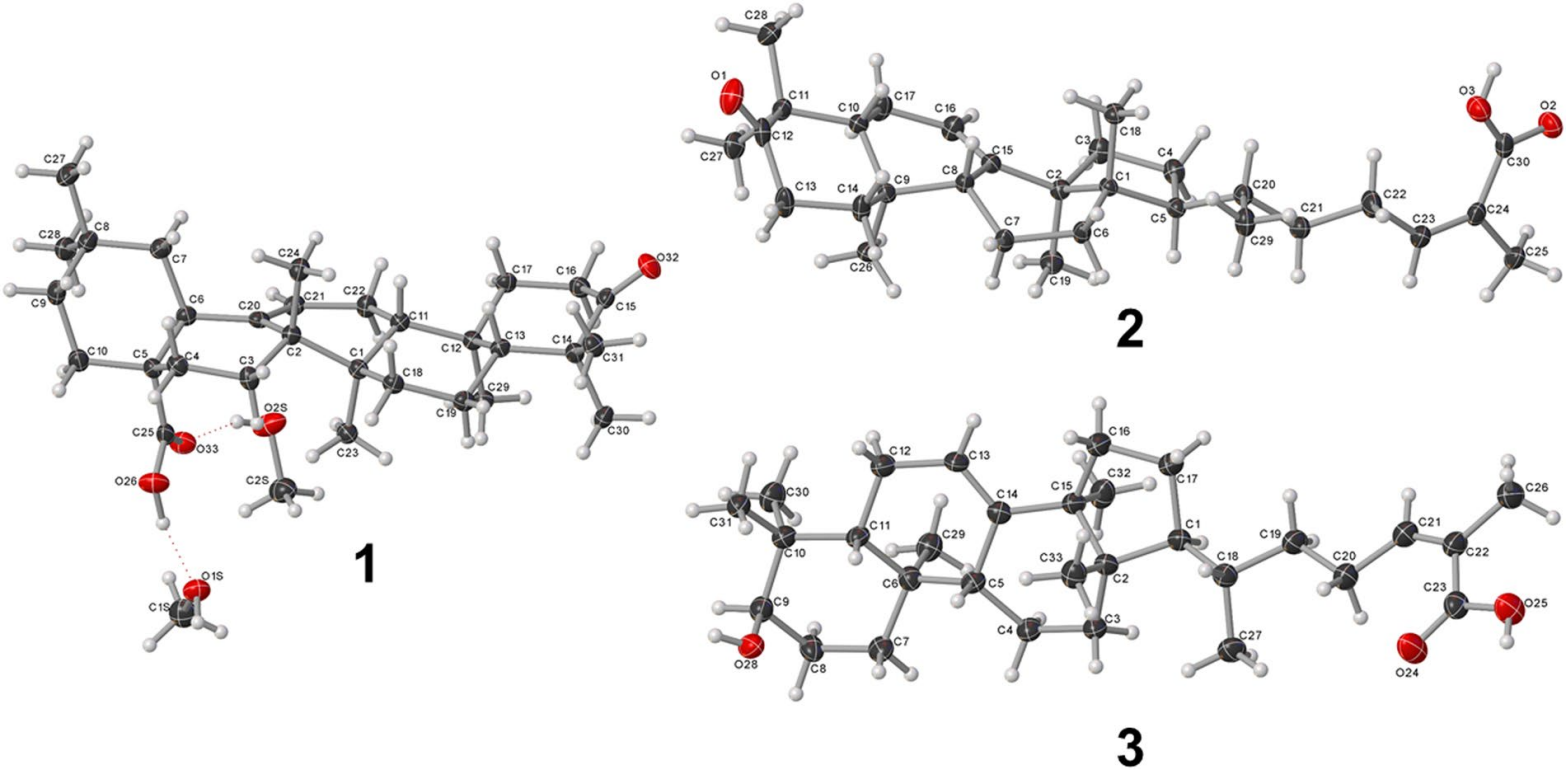
OLEX2 projections of compounds **1**-**3** (crystallographic numbering).

Structure determination was performed using Nuclear Magnetic Resonance (NMR) (Supplementary Figs. 2–4) and Mass Spectrometry (MS) (Supplementary Figs. 5–7). The compounds were identified as 3-oxo-olean-12-en-28-oic acid (**1**)^26^, 3-oxotirucalla-7,24*Z*-dien-26-oic acid (**2**)^27^ and 3α-hydroxytirucalla-7,24*Z*-dien-26-oic acid (**3**)^28^ (Fig. 1).The structures of **1**-**3** were further confirmed by single-crystal, X-ray diffractometry (**Supplementary.cif files provided**) with the assignment of the absolute configuration at the chiral centers (Fig. 2).

### Spectroscopic data for Compounds 1–3

3-oxo-olean-12-en-28-oic acid (**1**). Crystal solid. ^1^H NMR (600 MHz, CD_3_OD): δ 5.27 (1 H, t, *J* = 3.7 Hz, H-12), 2.87 (1 H, dd, *J* = 4.6, 14.0 Hz, H-18), 2.56 (1 H, dt, *J* = 8.0, 10.7 Hz, H-2β), 2.38 (1 H, m, H-2α), 1.18 (3 H, s, H-27), 1.08 (3 H, s, H-23), 1.07 (3 H, s, H-25), 1.05 (3 H, s, H-24), 0.95 (3 H, s, H-30), 0.91 (3 H, s, H-29), 0.88 (3 H, s, H-26); HRMS-APCI *m/z* 453.3370 [M-H]^-^ (calcd for C_30_H_44_O_3_, 453.3374).

3-oxotirucalla-7,24*Z*-dien-26-oic acid (**2**). Crystal solid. ^1^H NMR (600 MHz, CD_3_OD): δ 5.92 (1 H, m, H-24), 5.34 (1 H, m, H-7), 2.84 (1 H, d, *J* = 5.5 Hz, H-2), 1.87 (3 H, s, H-27), 1.13 (3 H, s, H-19), 1.05 (3 H, s, H-29), 1.03 (6 H, s, H-28 and H-30), 0.92 (3 H, d, *J* = 6.5 Hz, H-21), 0.85 (3 H, s, H-18), HRMS-APCI *m/z* 453.3369 [M-H]^-^ (calcd for C_30_H_45_O_3_, 455.3374).

3α-hydroxytirucalla-7,24Z-dien-27-oic acid (**3**). Amorphous solid. ^1^H NMR (600 MHz, CDCl_3_): δ 6.08 (1 H, t, *J* = 7.5 Hz, H-24), 5.25 (1 H, m, H-7), 3.46 (1 H, t, *J* = 2.9 Hz, H-3), 1.91 (3 H, s, H-27), 0.97 (3 H, s, H-28), 0.94 (3 H, s, H-30), 0.91 (3 H, s, H-29), 0.89 (3 H, s, H-21), 0.82 (3 H, s, H-18), 0.77 (3 H, s, H-19); HRMS-APCI *m/z* 455.3525 [M-H]^−^ (calcd for C_30_H_47_O_3_, 455.3531).

### X-ray Crystallographic Analysis of Compounds 1-3

Crystallographic data for compounds **1**-**3** have been deposited with the Cambridge Crystallographic Data Centre, 12 Union Road, CB2 1EZ, UK (fax: +44-1223-336033; e-mail: deposit@ccdc.cam.ac.uk) and are available on request quoting the deposition number CCDC: 1949041 (compound **1**), 1949042 (compound **2**) and 1949043 (compound **3**).

Crystallographic data of **1**. (C_30_H_46_O_3_). 2 (CH_4_O), *M*_*r*_ = 518.75, monoclinic, *I*2 (No. 5), a = 11.8169 (3) Å, b = 7.3667(2) Å, c = 34.0413(12) Å, *β* = 95.459(3), *α* = *γ* = 90°, *V* = 2949.88(16) Å^3^, *T* = 106 (6) K, *Z* = 4, *Z*’ = 1, *μ*(CuK_*α*_) = 0.601 mm^-1^, 14060 reflections measured, 5225 unique (*R*_*int*_ = 0.0516) which were used in all calculations. The final *wR*_*2*_ was 0.1073 (all data) and *R*_*1*_ was 0.0403 (I > 2σ(I)).

Crystallographic data of **2**. C_30_H_46_O_3_, *M*_*r*_ = 454.67, monoclinic, *P*2_1_ (No. 4), a = 6.81969(10) Å, b = 19.7846(3) Å, c = 19.4400(3) Å, *β* = 94.4735(13)^°^, *α* = *γ* = 90^°^, *V* = 2614.95(7) Å^3^, *T* = 107(8) K, *Z* = 4, *Z*’ = 2, *μ*(CuK_*α*_) = 0.557 mm^-1^, 29953 reflections measured, 10230 unique (*R*_*int*_ = 0.0408) which were used in all calculations. The final *wR*_*2*_ was 0.0939 (all data) and *R*_*1*_ was 0.0350 (I > 2σ(I)).

Crystallographic data of **3**. (C_30_H_48_O_3_). (CH_4_O), *M*_*r*_ = 488.72, orthorhombic, *P*2_1_2_1_2_1_ (No. 19), a = 7.43165(12) Å, b = 12.8741(2) Å, c = 59.8281(12) Å, *α* = *β* = *γ* = 90^°^, *V* = 5724.12(18) Å^3^, *T* = 102(2) K, *Z* = 8, *Z’* = 2, *μ*(CuK_*α*_) = 0.564, 61328 reflections measured, 10360 unique (*R*_*int*_ = 0.1169) which were used in all calculations. The final *wR*_*2*_ was 0.1301 (all data) and *R*_*1*_ was 0.0503 (I > 2σ(I)).

### Triterpenoid acids 1-3 exhibit limited growth inhibitory activity in *S. aureus* strains

**Figure 3:**
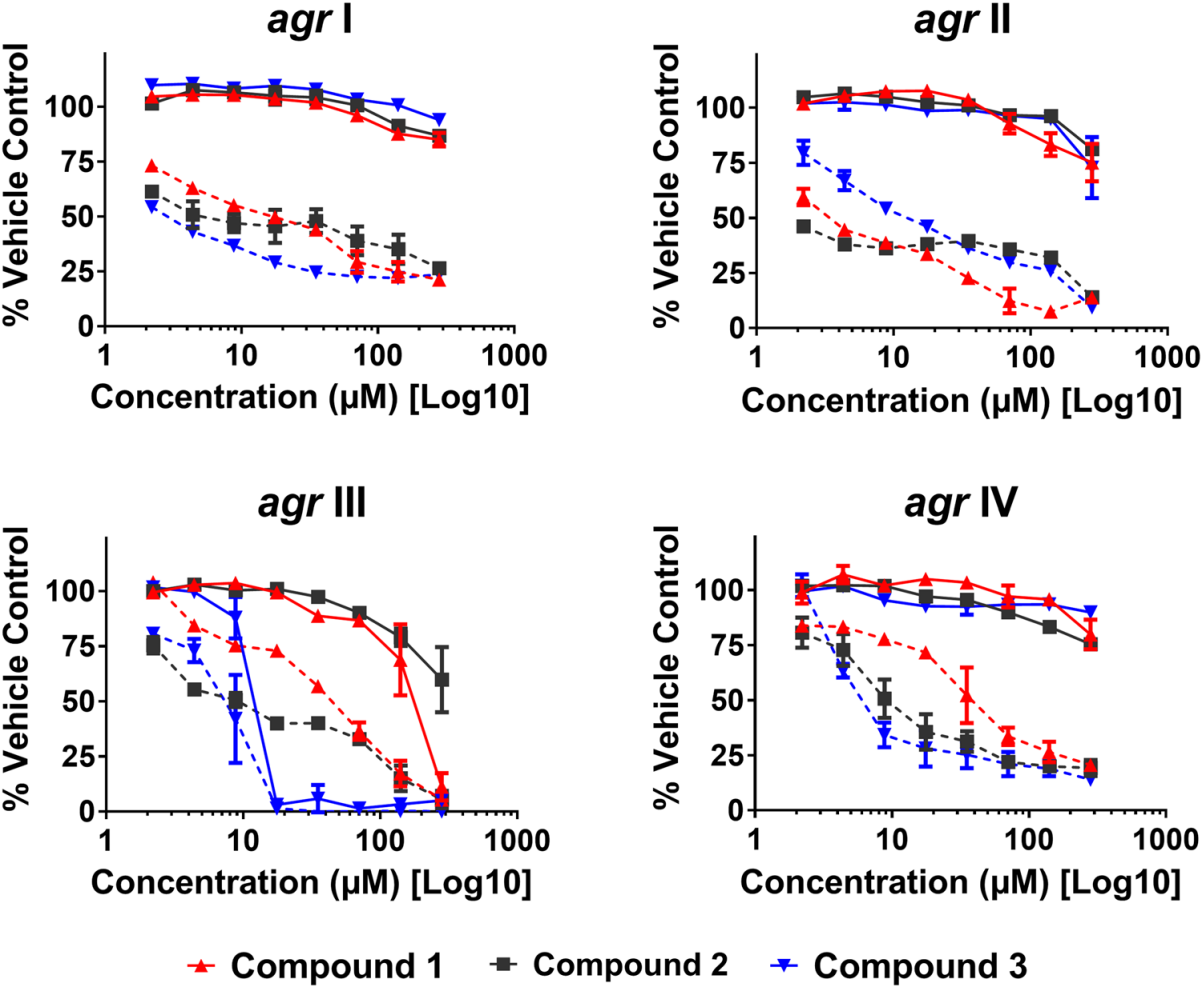
*Schinus* compounds 1-3 inhibit *agr* activity for all four *agr* alleles in a dose-dependent manner. Data are represented as percent *agr* activity or growth of the vehicle (DMSO) control at 24 hours against the following strains: AH1677 (*agr* I), AH430 (*agr* II), AH1747 (*agr* III), and AH1872 (*agr* IV). The dashed lines represent *agr* activity, measured by fluorescence, and the solid lines represent growth, measured using OD_600_. Significant differences (P < 0.05) in quorum sensing activity between the vehicle control and compounds **1**-**3** were observed at all doses tested in *agr* groups I, II and IV, with exception of the lowest concentration tested (2 µM) of **1** in *agr* III and **3** in *agr* IV.

Compounds **1**-**3** from *S. terebinthifolia* were screened against eight *S. aureus* strains at the test range of 4-563 µM in broth microdilution assays. Growth inhibition by dose response of the *agr* reporter strains is reported in Fig. 3, with a summary of MICs on all tested strains reported in Table 1. None of the three compounds exhibited an IC_50_ (≥50% growth inhibition in comparison to vehicle control) and MIC (≥90% inhibition) values for MRSA *agr* group I (AH1677), II and IV reporter strains. With regards to MRSA *agr* group III, Compound **1** exhibited weak growth inhibition (~15% inhibition) at up to 80 µM, but reached an IC_50_ by 141 µM and MIC at 281 µM, leaving a small gap between growth and quorum sensing inhibitory activity (QS IC_50_: 141 µM; IC_00_: 281 µM). Compound **3** exhibited stronger growth inhibitory activity against this *agr* group III reporter strain, reaching an MIC at 18 µM. Compounds **2** and **3** exhibited similar growth inhibitory activity against UAMS-929 and UAMS-1, IC_50_ and MIC of 35 and 70 µM, respectively. Also, **2** and **3** inhibited the growth of MRSA *agr* group I (AH1263, NRS249) with MICs of 563 and 18 µM, respectively for **2** and MICs of 141 and 281 µM for **3**. Importantly, MICs for AH1263 (LAC, the *agr* group I USA300 strain used in the murine model of dermonecrosis) were not detected at concentrations of up to 563 µM for compound **1**, and were noted at the higher concentrations of 563 and 141 µM for compounds **2** and **3**, respectively.

**Table 1:** MICs (μM) of *S. terebinthifolia* compounds **1-3** on *S. aureus*. Extract 430F-F5 is reported as μg/mL.

Strain	430F-F5		Compound **1**		Compound **2**		Compound **3**	
	IC_50_	MIC	IC_50_	MIC	IC_50_	MIC	IC_50_	MIC
AH1263; LAC (*agr* I)	ND	ND	ND	ND	141	563	35	141
NRS249 (*agr* I)	ND	ND	ND	ND	9	18	18	281
AH1677 (*agr* I)	ND	ND	ND	ND	ND	ND	ND	ND
AH430 (*agr* II)	ND	ND	ND	ND	ND	ND	ND	ND
AH1747 (*agr* III)	ND	ND	141	281	ND	ND	9	18
AH1872 (*agr* IV)	4	32	ND	ND	ND	ND	ND	ND
UAMS-929	−	−	ND	ND	35	70	35	70
UAMS-1	128	ND	ND	ND	35	70	35	70

Note: “ND“: MIC not detected at the test range (4 – 563 μM).

### Triterpenoid acids (1-3) inhibit quorum sensing and toxin production

To identify small molecule inhibitors of *S. aureus* quorum sensing from the 430F-F5 fraction, we employed a bioassay-guided fractionation strategy using *agr*-fluorescent reporter stains that represent each of the four known *agr* allelic groups (Fig. 3, Supplementary Table 1). Strains were grown in the presence of the compounds and monitored for growth inhibition by optical density and *agr* activity by fluorescence.

Compounds **1-3** significantly inhibited quorum sensing in all four *agr* types, exhibiting IC_50_ values ranging from 4–70 µM for **1**, 2–9 µM for **2**, and 4–9 µM for **3** (Fig. 3, Table 2). Compound **3** showed the most potent quorum sensing inhibition activity in this model (IC_90_ of 18 µM), but this was influenced by high growth inhibition of the *agr* III reporter by this compound. With the exception of this case (compound **3** growth inhibition of the *agr* III reporter strain), **1**-**3** exhibited limited growth inhibitory activity against the reporter strains.

**Table 2:** IC_50_ and IC_90_ (μM) for quorum sensing inhibitory activity of *S. terebinthifolia* compounds **1-3** on *S. aureus*.

Strain	Compound 1		Compound 2		Compound 3	
	IC_50_	IC_90_	IC_50_	IC_90_	IC_50_	IC_90_
AH1677 (*agr* I)	18	ND	4	ND	4	ND
AH430 (*agr* II)	4	70	2	281	9	281
AH1747 (*agr* III)	70	281	9	281	9	18
AH1872 (*agr* IV)	35	ND	9	ND	9	281

Note: “ND“: Value not detected at the test range (2 – 281 μM).

**Figure 4:**
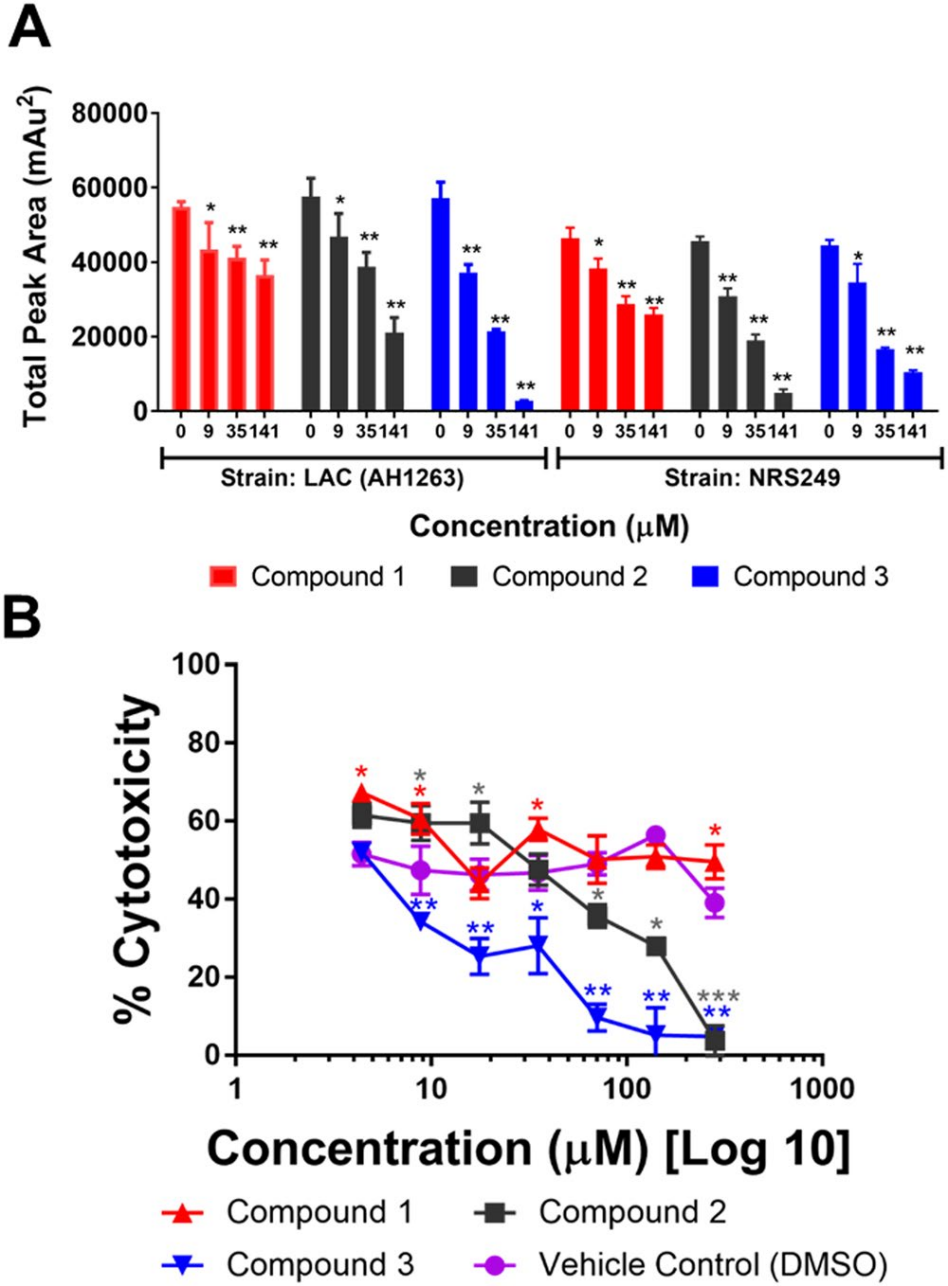
Compounds 1-3 inhibit δ-toxin production in a dose-dependent manner. (**A**) Levels of δ-toxin were quantified by HPLC analysis of culture supernatant following treatment with sub-MIC50 concentrations of compounds **1-3**. (**B**) General toxicity of *S. aureus* supernatants to HaCaTs. An immortalized line of human keratinocytes was treated with supernatants of *S. aureus* (NRS249) that were grown + /− compounds **1**-**3** or vehicle (DMSO). Statistical significance by ANOVA in comparison to the vehicle treated wild type control is denoted as *P < 0.05, **P < 0.01, ***P < 0.001. Error bars represent mean and SEM.

To verify the observed quorum-inhibition effects of **1**-**3**, we assessed their capacity to inhibit δ-toxin production at concentrations ranging from 9 to 141 µM. High level δ-toxin producing of strains of *S. aureus* were treated with the triterpenoid acids and the supernatants harvested for RP-HPLC and human keratinocyte cell (HaCaT) toxicity analysis. Each of compounds **1-3** were effective in significantly reducing δ-toxin production, with weak or no growth inhibition in the *S. aureus* strains examined (Fig. 4A).

To assess any remaining virulence factors linked to cytotoxicity (e.g., phenol soluble modulins, PSMs), sterile filtered supernatants of NRS249 cultures grown with compounds **1**-**3**, were exposed to an immortalized line of human keratinocytes (HaCaTs) and assessed for damage by lactate dehydrogenase (LDH) assay. The toxicity of *S. aureus* supernatants to HaCaTs cells was inhibited in a dose dependent manner (Fig. 4B).

To examine the specificity of **1-3** against *agr*, we also chose reporter strains (promoter-GFP fusions in the *Staphylococcus aureus* AH1263 background) that are known to be either *agr-*regulated or independent of *agr* regulation.

**Figure 5:**
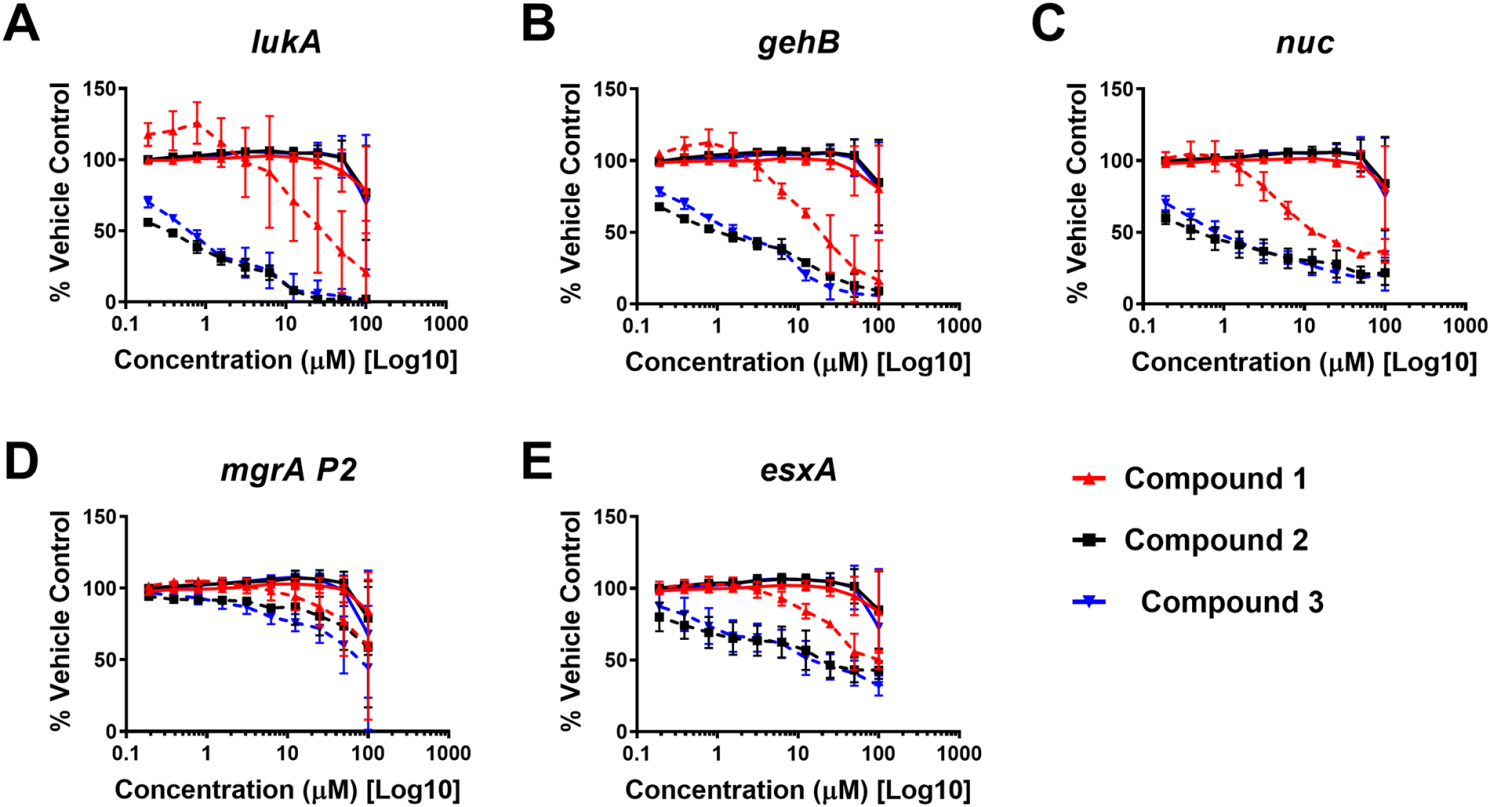
*Schinus* compounds 1-3 inhibit other classes of MRSA virulence factors in a dose dependent manner. Select MRSA virulence factor reporter strains were treated with increasing doses of *Schinus* compounds **1**-**3** and growth (OD_600_, solid lines) and reporter activity (GFP, dashed lines) were measured over 24 hours. The 24-hour time point of treated groups normalized to the DMSO-only control is shown. Results are pooled from 3 independent experiments and the mean + /− SD is shown.

The *agr*-regulated reporters (*lukA*: leukocidin A, *gehB*: glycerol ester hydrolase or lipase) showed dose-dependent inhibition by **1-3** as expected since we have shown that these compounds target the *agr* system (Fig. 5A,B).

To our surprise, the *nuc* reporter (nuclease), which is primarily under the control of the *sae* two-component system, is also inhibited in a dose-dependent manner by all of the *Schinus* compounds (**1-3**), suggesting broader inhibitory effects against multiple MRSA virulence factors (Fig. 5C). Additionally, we tested the *agr*-independent *mgrA P2* reporter (a constitutive promoter from the *mgrA* two-component system) and saw little effect of any *Schinus* compound (**1-3**), suggesting that these two components do not primarily target this system (Fig. 5D). We found for the *agr*-independent *esxA* reporter, which is a component of the MRSA type VII secretion system and generally thought to be under the control of the *mgrA* two-component system (but may be regulated by other systems as well), that there was modest inhibition by **1-3** (Fig. 5E).

Taken together, these results confirm that 1-**3**are potent *agr* inhibitors but are also broader MRSA virulence inhibitors that may also target the *sae* two-component system.

### Triterpenoid acids 1-3 impact biofilm formation

**Figure 6:**
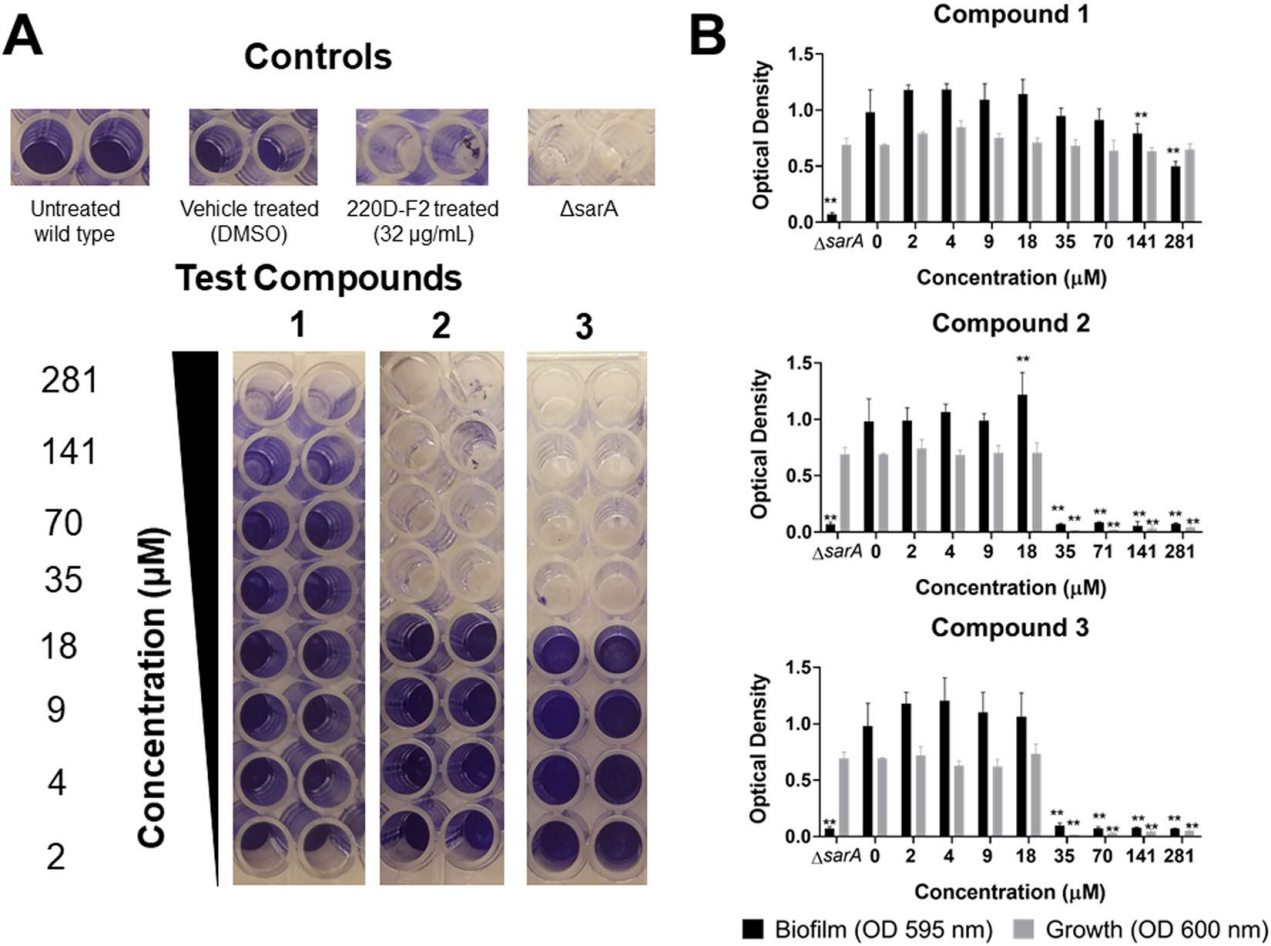
Impact of compounds 1-3 on *S. aureus* biofilm formation and planktonic growth in a biofilm model. USA 200 isolate UAMS-1 and its isogenic *sarA* mutant (UAMS-929) were used in the biofilm assay. (**A**) Images of crystal violet stained biofilm in 96-well plates. (**B**) The optical density of the crystal violet eluent from the adherent biofilm (OD 595 nm) is plotted along with the optical density for the planktonic cells (OD 600 nm), measured by transfer of the well supernatants to a new 96-well plate. Statistical significance by ANOVA in comparison to the vehicle treated wild type control is denoted as **P < 0.05, **P < 0.01, ***P < 0.001. Error bars represent mean and SEM.

In microtiter plate biofilm assays, compound **1** increased biofilm formation at low dosages (2–70 µM), and reduced biofilm at higher concentrations (141– 281 µM) without growth inhibition. This is noteworthy as the neither the growth inhibitory IC_50_ or MIC for **1** in the biofilm strain (UAMS-1) was detected at a maximum test concentration of 563 µM (Table 1). Compounds **2** and **3** caused significant, dose-dependent increases in biofilm biomass at concentrations ranging from 2–16 μM. In contrast, both biofilm formation and bacterial growth were strongly reduced at concentrations of 35 μM and above (Fig. 6). For compounds **2** and **3**, these biofilm inhibitory concentrations are roughly 4-18 times higher than the IC_50_ for quorum sensing inhibition in the four reporter strains (Table 2).

### Impact of compounds 1-3 on human keratinocytes

**Figure 7:**
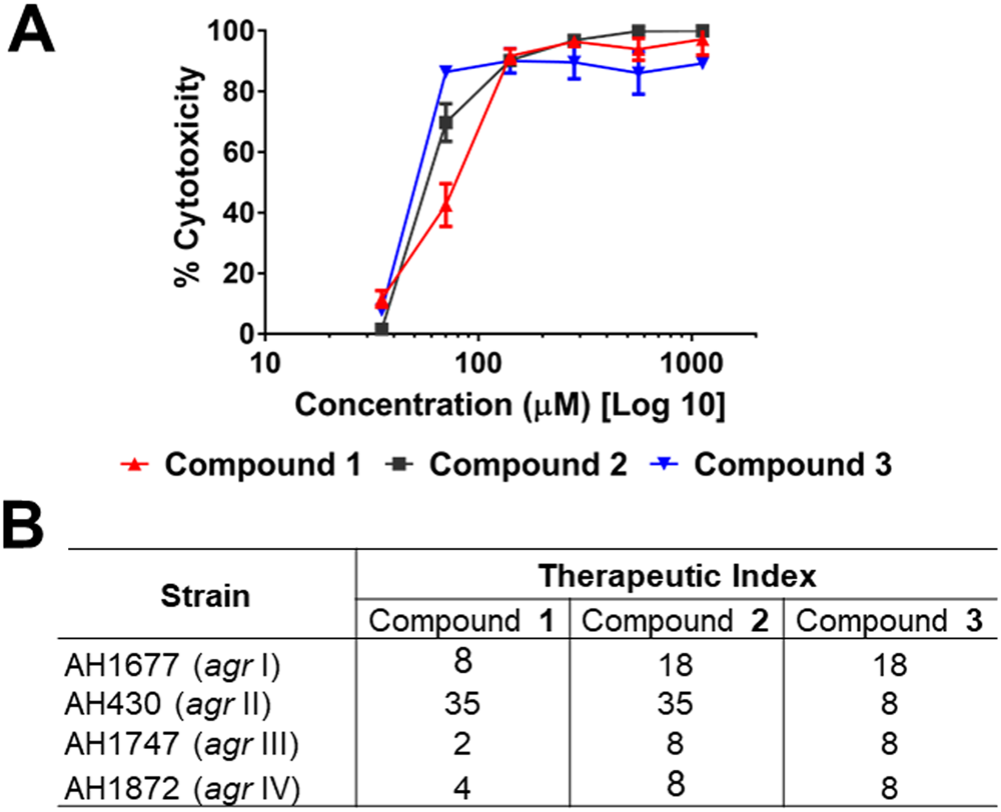
Impact of compounds on quorum sensing inhibition in comparison to cytotoxicity in a human keratinocyte cell line for calculation of the therapeutic index. (**A**) Cytotoxicity of compounds **1**-**3** on human keratinocyte (HaCaT) cell line by LDH assay for cell viability, with IC_50_ of 140 µM for **1** and 70 µM for **2** and **3**. (**B**) The therapeutic index for each compound is calculated as IC_50_ for HaCaT cytotoxicity divided by the IC_50_ for *agr* activity (see Table 2) in each reporter strain.

To determine the potential toxicity to human cells, compounds **1**-**3** were tested on human keratinocyte cells (HaCaTs) using a lactate dehydrogenase (LDH) assay for cytotoxicity. The IC_50_ values for cytotoxicity to HaCaTs and therapeutic indices for each reporter strain are reported in Fig. 7. The therapeutic index ranged from 2 to 35, depending on the reporter strain and compound combination.

### Compounds 1-3 abate quorum sensing and dermonecrosis *in vivo*

**Figure 8:**
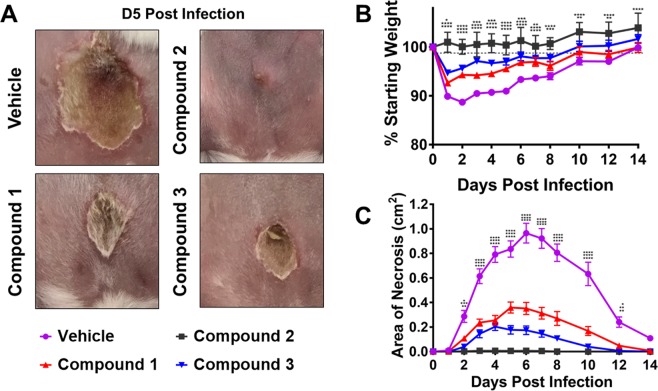
Compounds 1-3 inhibit MRSA quorum sensing and protect skin from dermonecrosis in a murine model of skin and soft tissue infection. (**A**) BALB/c mice were intradermally challenged with 1×10^8^ CFUs of MRSA (LAC) and 50 µg of respective compound or the vehicle control (DMSO). A representative image of the dermo-necrotic lesion is shown for Day 5 post-infection. (**B**) Compounds **1**-**3** significantly reduce morbidity as measured by animal weight loss over time compared to vehicle control. (**C**) A single 50 µg dose of each *Schinus* compound significantly attenuates MRSA-mediated dermatopathology as measured by lesion size. Significant differences between vehicle and compounds **1**-**3** were determined by Ordinary 2-way ANOVA with Dunnett’s correction for multiple comparisons. *P < 0.05, **P < 0.005, ***P < 0.0005, ****P < 0.0001. Error bars represent mean and SEM.

The antivirulence activity of compounds **1-3** observed *in vitro* led us to assess their efficacy as an *in vivo* anti-infective. Using a previously established murine model of dermo-necrosis^15,25,29^, BALB/c mice were intradermally challenged with MRSA (USA300) and 50 µg of each *Schinus* compound or DMSO vehicle control at the time of infection. Over the two-week course of infection, compounds **1** and **3** exhibited similar potency in reducing MRSA-mediated skin damage (Fig. 8). Compound **2** effectively reduced the dermonecrotic lesion area to nearly undetectable levels (Fig. 8A**)**. All compounds significantly minimized animal weight loss, indicating decreased morbidity compared to wild-type infection (Fig. 8B). Additionally, each compound significantly reduced dermonecrotic lesion size throughout the course of MRSA infection (Fig. 8C**)**. Taken together, these results demonstrate that all of the compounds protect mice from MRSA infection-associated dermonecrotic injury and morbidity.

## Discussion

The emergence of antibiotic resistance, together with the lack of antibiotics based on novel molecular scaffolds, marks the entry to the ‘post antibiotic era’. Interference with bacterial virulence has emerged as an attractive approach among the current strategies for developing new anti-infective drugs. Prior studies have reported the capacity of plant extracts and phytochemicals to interfere in intra and inter-species quorum sensing communication systems^30,31^. The ability of plants to interrupt quorum sensing systems may serve as a defense mechanism to fight against bacterial invasion. One of the keys factors of success of phytochemicals could be their similitude to what is considered the ideal quorum sensing inhibitor, which includes being chemically stable, highly effective low-molecular-mass molecules and harmless for human health^32^.

In a previous study, we confirmed the quorum sensing activity of a refined *S. terebinthia* fruit extract (430D-F5) against multiple MRSA strains^25^. Here, we successfully isolated three triterpenoid acids using a bioassay-guided fractionation approach. The structure of these compounds (**1**-**3**) was determined via NMR, MS, and X-ray crystallography, and matches with substances previously isolated from *S. terebinthifolia* and other plants^33–35^. The crystal structures of compounds **2** and **3** were previously reported^36,37^.

We found that the three triterpenoid acids (**1**-**3**) inhibited expression of all *S. aureus agr* types in a dose dependent manner with limited inhibition of bacterial growth in *agr* groups I, II, and IV, but moderate growth inhibition in the *agr* group III reporter. Compound **3** showed the most potent anti-virulence activity against *S. aureus in vitro* (Table 2, Fig. 3), while compound **2** exhibited the greatest level of protection from dermonecrosis *in vivo*. To confirm the inhibitory activity of isolated compounds with a downstream protein level readout, we assessed their ability to inhibit the production of *agr*-regulated δ-toxin. These triterpenoid acids (**1**-**3**) notably decreased δ-toxin production in two *S. aureus* strains, and the general toxicity of pre-treated *S. aureus* supernatants to HaCaTs also confirmed these effects.

The impact on other *agr-*regulated reporters were also assessed, revealing dose-dependent inhibition of leucocidin A *(lukA*) and glycerol ester hydrolase or lipase (*gehB*). Little to no effect of any compound was noted for the *agr-*independent *mgrA P2* or *esxA* reporters. Interestingly, a dose-dependent response was noted in tests with the nuclease reporter, which is under the control of the *sae* two-component system, suggesting a dual role of **1**-**3** in broader virulence inhibition across both *agr* and *sae* systems.

The *agr* operon is essential for virulence-factor production during MRSA skin-infection and reducing *agr* functionality could potentially mitigate acute tissue damage. This is supported by the results of our *in vivo* experiments, where **2** decreased MRSA-associated tissue damage to the limit of detection. Notably, **1** and **3** also exhibited significant anti-virulence effects, and mice were protected from severe dermonecrosis and morbidity. However, it is well-established that the absence of *agr* signaling drives MRSA biofilm formation under *in vitro* conditions^38^. Indeed, low concentrations of all compounds resulted in significant biomass accumulation while higher concentrations mitigated biomass accumulation in our *in vitro* assays. These results suggest that careful titration of quorum sensing inhibitory compound doses will certainly be necessary if administered in a human infection scenario. Additionally, this study employed inhibitory compounds at the onset of infection in a prophylactic model, effectively reducing MRSA virulence during infection initiation and allowing for rapid clearance of bacteria by the immune system. The use of quorum sensing inhibitors at the onset of human infection is far less likely if disease progression is slow or access to healthcare providers is limited. Thus, we postulate that natural product quorum sensing inhibitors, such as those proposed in this study, could be paired with a secondary bactericidal or biofilm dispersal agents to fully abrogate MRSA infection. However, further research is needed to identify antibiotics that synergize with virulence inhibitory compounds while mitigating the risk of acquired MRSA resistance^39^.

Our results also suggest that the biological activity of these compounds may be particularly sensitive to chemical modifications of their terpenoid skeleton. In particular, this is the case for compounds **2** and **3**, where the substituent at carbon 3 could play a key role in the inhibition of virulence factors. A medicinal chemistry approach to modification of the terpenoid skeleton could also be explored to improve the therapeutic indices of these compounds, with an optimal difference between activity and toxicity being several hundredfold. This will require study of a large number of related compounds in order to approach the minimum requirements necessary for reaching a more definitive conclusion with respect to structure-activity relationships (SAR).

Previous studies have reported related pentacyclic triterpenoids with anti-virulence and biofilm formation/eradication activities against *S. aureus* and other strains, such as betulinic acid^40^, asiatic and corosolic acids^41^, ursolic acid and derivatives^42,43^, glycyrrhetinic acid^44^ and oleanolic acid^40^. However, to the best of our knowledge, this is the first time that the anti-quorum sensing activity of tirucallane-type triterpenoids (**2** and **3**) is described.

## Materials and Methods

### Plant material

*Schinus terebinthifolia* Raddi, Anacardiaceae fruits were collected in bulk from private lands in DeSoto County, Florida in November of 2013 and 2014 after obtaining permission from the land owner. Vouchers were deposited at the Emory University Herbarium (GEO) (Voucher CQ-400, GEO Accession No. 020063) and were identified using the standard Flora for Florida. Plant material was then dried in a desiccating cabinet at low heat. Once dry, plant material was sealed in paper bags and stored at room temperature until further processing.

### Extraction and bioassay guided fractionation

Fruits of *S. terebinthifolia* were dried in a dehumidifying chamber, ground into a powder with a Wiley Mill at 2 mm mesh and extracted using an ultrasonic bath at room temperature with methanol (ratio of 1 g dry material: 10 mL MeOH) for a total of three times. Filtered extracts were combined, concentrated at reduced pressure with rotary evaporators (<40 °C), and lyophilized. The crude extract (15.0 g) was suspended in 400 mL methanol-water (1:4) solution and underwent sequential liquid-liquid partitioning with hexane (3×500 mL), ethyl acetate (3×500 mL), and n-butanol (3×500 mL). The combined partitions were evaporated to dryness to yield 1.16 g (3.92%) of hexane residue, 1.15 g (2.98%) of ethyl acetate residue and 12.61 g (32.71%) of aqueous residue.

Following initial quorum sensing assays, the most active partition, the aqueous residue (430 F) was subjected to fractionation through flash chromatography. Fractionation was performed using a CombiFlash® Rf+ Lumen (Teledyne ISCO) flash chromatography system with a RediSep Rf Gold silica column. The dry load column was prepared by binding 430 F to Celite 545 at a ratio of 1:4. Flash chromatography was performed using a three-solvent system of (A) hexane, (B) dichloromethane, and (C) methanol. The gradient began with 100% A for 6 column volumes (CV), then went to 100% B over 12 CV and was held for 18.2 CV. The gradient was then changed to 74.5:25.5 B:C over the course of 3.1 CV. These conditions were held for 6.8 CV, following which the gradient changed to 68.8:31.2 B:C over 0.7 CV and was held at these conditions for 7.5 CV. Finally, the gradient was adjusted to 100% C over 2.2 CV and held for 14.6 CV. The chromatography was monitored at 254 and 280 nm, as well as via evaporative light scanning detector (ELSD). Tube volumes (25 mL each) were combined to create eight fractions 430F-F1 (tubes 1–10), 430F-F2 (tubes 11-17), 430F-F3 (tubes 18-27), 430F-F4 (tubes 28-42), 430F-F5 (tubes 44-45), 430F-F6 (tubes 46-62), 430F-F7 (tubes 63-71), 430F-F8 (tubes 72-82). 430F-F5 was the most active fraction after testing for quorum sensing activity.

430F-F5 was further fractionated by preparative HPLC (Prep-HPLC) [Agilent 1260 infinity II LC system (CA, USA) equipped with a UV-vis detector, auto collector, Agilent XDB-C18 (30 × 250 mm, 5 μm) column, eluted with A: H_2_O, B: MeOH at 42.5 mL/min using the following gradient: 0 min 50% B, 0-11.5 min 50% B, 11.5-36.5 min 100% B, 36.5-46.5 min 100% B, detection at 254 and 320 nm)] to afford 12 sub-fractions. 430F-F5-PF11 and 430F-F5-PF12 showed the highest activity for the inhibition of quorum sensing.

430F-F5-PF11 and 430F-F5-PF12 were fractionated/purified using the same prep-HPLC system described above and eluted with A: H_2_O, B: Acetonitrile at 42.5 mL/min using the following gradient: 0 min 90% B, 12 min 95% B, 30 min 95% B. From 430F-F5-PF11 were obtained compound **1** (100.0 mg). From 430F-F5-PF12 were isolated compound **2** (178.1 mg) and **3** (52.0 mg).

### Analytical chemistry procedures

Nuclear magnetic resonance (NMR) data, ^1^H NMR (600 MHz) were measured on a Bruker AVANCE III HD 600 (600 MHz for ^1^H-NMR, 5 mm CryoProbe) spectrometer in CD_3_OD or CDCl_3_ solvents. Chemical shifts (δ) are reported in ppm with the solvent peaks used as reference.

High resolution/accurate mass-Atmospheric pressure chemical ionization (HRMS-APCI) experiments were performed on a Thermo Exactive Plus using the Ion Max Source with APCI probe. The sample was placed on a melting point capillary and then placed in an Ion Sense ASAP (Atmospheric Solid Analysis Probe) probe, which was inserted into the source to enable gas from the APCI probe to blow on the capillary tube. The APCI probe uses nitrogen gas at arbitrary units of 50 and was heated to a temperature of 450 °C. A discharge on the needle with a discharge current of 5 µA was used to ionize the sample, with a capillary temperature of 320 °C, S-lens RF level 70 and an AGC setting of 1 E-6. The maximum injection time was 50 ms. Spectra were collected with 140,000 resolution at *m/z* 200 using Tune software and analyzed with Thermo’s Freestyle software.

Single crystals of compounds **1**-**3** were recrystallized from methanol by placing them into a −20 °C freezer overnight. A suitable crystal was selected and the crystal was mounted on a Rigaku XtaLAB Synergy-S diffractometer. The crystal was kept at 107(8) K during data collection. Using Olex2, the structure was solved with the ShelXT structure solution program using Intrinsic Phasing and refined with the ShelXL refinement package using Least Squares minimization.

### Bacterial strains

Quorum sensing reporter strains of *S. aureus* representing the four *agr* groups included AH1677, AH430, AH1747 and AH1872, were used. AH1677 is an *agr* I reporter from strain AH845 (CA-MRSA USA 300 LAC), AH430 is an *agr* II reporter from SA502A, AH1747 is an *agr* III reporter from strain CA- MRSA MW2, and AH1872 is an *agr* IV reporter from MN EV. All strains contained plasmid pDB59 that served as an *agr* fluorescence reporter and was maintained through culture in media containing chloramphenicol at a concentration of 10 μg mL^−1^. Chloramphenicol (Sigma-Aldrich) was dissolved in 95% EtOH to a stock concentration of 10 mg mL^−1^ and stored at −20 °C until being added to the media. Cultures were grown on Tryptic Soy Agar (TSA) supplemented with 10 μg mL^−1^ chloramphenicol. Two *S. aureus* strains (UAMS-1, UAMS-929) served as biofilm test strains, with the latter being an isogenic *Δsar* mutant that has a limited capacity to produce a biofilm. One *S. aureus* strain (NRS385) was used for δ-toxin quantification experiments. UAMS-1, UAMS-929 and NRS385 were grown in Tryptic Soy Agar (TSA) and Tryptic Soy Broth (TSB). A summary of strain characteristics is provided in Supplementary Table 1.

### Minimum inhibitory concentration

Fraction 430F-F5 and isolated compounds of *S. terebinthifolia* **1**-**3**, were evaluated for minimum inhibitory concentrations (MICs) against the four *agr* groups, the biofilm test strain (UAMS-1) and NRS249 (for δ-toxin quantification experiments) following the Clinical Laboratory Standards Institute (CLSI) M100-S23 guidelines for microtiter broth dilution testing^45^. Fraction, compounds **1**-**3** and vehicle were tested at a concentration range of 4 to 563 μM, using 2-fold serial dilution. The optical density (OD_600nm_) was measured with a BioTek Cytation 3 multimode plate reader, to calculate percent inhibition of growth. The IC_50_ for growth was defined as the lowest concentration at which an extract displayed ≥ 50% inhibition and MIC (IC_90_) at ≥ 90% inhibition. Control included the vehicle (DMSO) and antibiotic (Ampicillin, MP Biomedicals Inc). All tests were performed in triplicate and repeated using a new stock of bacteria on a separate day.

### Agr reporter assay for quorum sensing inhibition

*S. aureus agr* reporter strains AH1677 (*agr* I), AH430 (*agr* II), AH1747 (*agr* III) and AH1872 (*agr* IV) were grown overnight in TSB supplemented with chloramphenicol at 37 °C while shaking at 200 rpm. Cultures of *S. aureus* were standardized by optical density (OD_600nm_) matching to a 0.5 McFarland standard to reach a final inoculum density in the wells of 5 × 10^5^ CFU mL^−1^ in TSB, supplemented with 10 μg/mL chloramphenicol. Compounds **1**-**3** were tested to escalating concentrations (2-281 μM) of each compound. Black sided, 96-well, clear bottom, tissue-culture treated plates (Costar 3603) with final well volume of 200 μL were used for all *agr* inhibition assays. Plates were incubated in a humidified chamber at 37 °C while shaking at 260 rpm. After 24 hours, plates were removed and OD_600_ and fluorescence were measured by plate reader at an excitation of 493 nm and emission of 535 nm. All tests were performed in triplicate and repeated using a new stock of bacteria on a separate day.

### MRSA virulence factor reporter assays

For reporter assays, overnight (ON) cultures of MRSA reporter strains (AH5116^46^, pCM29-P*lukA*, Cm^R^; AH5382^46^, pCM29-P*gehB*, Cm^R^; AH3613^47^, *mgrA* P2-sGFP fusion, Erm^R^; AH5101^48^, P*nuc*-sGFP, Cm^R^; AH5095^46^, pCM29-P*esxA*, Cm^R^) were prepared by sub-culturing 1:500 in fresh TSB (5% DMSO) with chloramphenicol (10 µg/mL) or erythromycin (5 µg/mL). Compounds **1**-**3** were resuspended in DMSO, and then diluted fresh in TSB for each assay replicate. 200 µL of compound was added to a black 96-well, clear bottom, tissue-culture treated plates (Costar) and diluted 2-fold in TSB (100 µM - 0.2 µM final compound concentrations). 100 µL of reporter culture was added to each well for 200 µL final volume. A vehicle control (TSB + DMSO only) was included for each reporter for each experimental replicate. Cultures were grown in a Stuart humidified incubator at 37 °C with shaking at 1000 RPM. At hourly time points, the plates were measured on a TECAN Group Ltd. Infinite 200 Pro plate reader to quantify growth (OD_600_ nm) and sGFP signal (Excitation: 480 nm, Emission: 515 nm).

### HaCaT cytotoxicity assay

The HaCaT cell line was maintained and cytotoxicity of compounds (1-3) were assessed using the LDH cytotoxicity assay as previously described^49^. The Therapeutic Index (TI) was calculated as a ratio of the TD_50_ (Toxic dose for cytotoxicity at IC_50_) and ED_50_ (effective dose for quorum sensing inhibiton IC_50_): = $\frac{{\rm{TD}}50}{{\rm{ED}}50}$. Cytotoxicity of supernatants was further evaluated using a viability/cytotoxicity assay and imaged with fluorescent microscopy as previously described^49^. All tests were performed in triplicate and the full experiment was repeated on a separate day using fresh cell stock.

### Quantification of δ-toxin

Levels of δ-toxin present in the culture supernatant of treated and untreated samples was quantified using NRS249 and AH1263 strains of *S. aureus* and following a previously described protocol^50^. Experiments were conducted in quadruplicate in 14 mL snap-cap tubes with a final volume of 1.5 mL, and repeated in full with fresh bacterial stock on a separate day. All compounds were tested at concentrations from 9-141 µM. Tubes were incubated at a 45° angle at 37 °C while shaking (275 rpm) for 15 hours. After incubation, cultures were placed on ice, an aliquot was taken to determine OD, then centrifuged at 13,000 rcf for 5 min at a temperature of 4 °C. Supernatants were removed and 750 μL of each supernatant was placed in a vial for HPLC quantification of δ -toxin. The remaining volume of supernatant was sterile filtered with a 0.22 μm nylon syringe filter and stored at −20 °C until needed for later treatment of cells in the HaCaT cytotoxicity assay. Level of δ-toxin in the bacterial supernatant treated and untreated with the compounds was quantified by RP-HPLC (Reversed-Phase High Performance Liquid Chromatography) as previously described^50^.

### Biofilm formation

All compounds were examined (2–281 μM) for impact on *S. aureus* biofilm formation using a human plasma protein-coated assay as previously described^49,51,52^. Additionally, to monitor for any growth impact in this assay, the optical density of planktonic cells in the biofilm wells was calculated by transferring the supernatant to a new 96-well plate and reading the OD_600nm_ in a BioTek plate reader. *S. aureus* isolate UAMS-1 was used for the biofilm assay, and its isogenic *sar*A mutant (UAMS-929), which has a biofilm deficient phenotype, served as a positive control. All tests were performed in triplicate and repeated using a new stock of bacteria on a separate day.

### Animal Studies

A previously described murine model of MRSA skin infection was used to determine the efficacy of each *Schinus* compound as an anti-infective^25^. All animal experiments described herein were approved by and conducted in accordance with the recommendations of the Animal Care and Use Committee at the University of Colorado Anschutz Medical Campus (IACUC protocol number 117217). One day prior to inoculation, the abdominal hair of 8 week old female BALB/c mice was shaved and chemically removed with topical application of Nair for 1 minute. LAC (USA300, AH1263) was grown in TSB media overnight at 37 °C in a shaking incubator (200 rpm). Overnight LAC culture was diluted 1:100 in fresh TSB media and allowed to grow to early log phase (~2 hr to OD_600nm_ 0.5). Cells were washed in sterile PBS and resuspended to achieve an inoculum of 1×10^8^ CFU. 50 μL inoculum suspensions containing 1×10^8^ CFU LAC and 50 μg of each compound **1**-**3** (diluted in DMSO) or DMSO vehicle control were injected intradermally into the abdominal skin of each BALB/c mouse. Baseline body weights for the mice were taken prior to injection and each day following. For determination of lesion size, digital photos of skin lesions were taken with an iPhone camera and analyzed with ImageJ software for Mac. Inoculum CFU was verified by serial dilution, plating, and colony counting after overnight incubation of the plate.

### Statistical Analyses

All data were analyzed using a two-tailed Student’s t-test as calculated by GraphPad Prism 7 software (GraphPad Software, La Jolla, CA). DMSO or dH_2_O treated (vehicle control) cultures were used as a vehicle control and were compared to those treated with extract for all statistical analyses. P < 0.05 were considered statistically significant. All assays and other experiments were performed in triplicate or quadruplicate to ensure sufficient technical replicates and repeated on two separate days to ensure sufficient biological replicates.

## Supplementary information

Supplementary Information.
