# Supplementary material for: Triterpenoid acids isolated from Schinus terebinthifolia fruits reduce Staphylococcus aureus virulence and abate dermonecrosis
Source: Sci Rep. 2020 May 15;10:8046. doi: 10.1038/s41598-020-65080-3 (PMC7229044; doi:10.1038/s41598-020-65080-3)

SUPPORTING INFORMATION

Triterpenoid acids isolated from *Schinus terebinthifolia* fruits reduce *Staphylococcus aureus* virulence and abate dermonecrosis

Huaqiao Tang[1,2†], Gina Porras[1†], Morgan M. Brown[3], Francois Chassagne[1], James T. Lyles[1], John Bacsa[4], Alexander R. Horswill[3], Cassandra L. Quave[1,5*]

[1]Center for the Study of Human Health, Emory University, 550 Asbury Circle, Candler Library 107E, Atlanta, Georgia, USA.
[2]Department of Pharmacy, Sichuan Agricultural University, Chengdu, Sichuan, China.
[3] Department of Immunology and Microbiology, University of Colorado School of Medicine, Aurora, CO 80045
[4] X-ray Crystallography Center, Emory University, 1515 Dickey Dr., Atlanta, GA 30322, USA.
[5] Department of Dermatology, Emory University School of Medicine, 615 Michael St., Rm 105L Whitehead Bldg, Atlanta, GA, USA.
[†] Co-first authors.

INDEX

Figure S1. Fractionation strategy, with percent yields of the active fractions and compounds. 2
Figure S2. ^{1}H NMR spectrum of compound **1**. 3
Figure S3. ^{1}H NMR spectrum of compound **2**. 4
Figure S4. ^{1}H NMR spectrum of compound **3**. 5
Figure S5. HRMS-APCI spectra of compounds **1-3**. 6
Figure S6. HRMS-APCI spectra of compounds **1-3**. 7
Figure S7. HRMS-APCI spectra of compounds **1-3**. 8
Figure S8. Quorum quenching activity step 4: fractions. 9
Figure S9. Quorum quenching activity step 5, 430F-F5-PF11: fractions and compounds. 10
Figure S10. Quorum quenching activity step 5, 430F-F5-PF12: fractions and compounds. 11
Table S1. Profile of bacterial strains used in this study. 12
Data Set 1. CheckCIF/PLATON report on compound **1**. 13
Data Set 2. CheckCIF/PLATON report on compound **2**. 17
Data Set 3. CheckCIF/PLATON report on compound **3**. 21

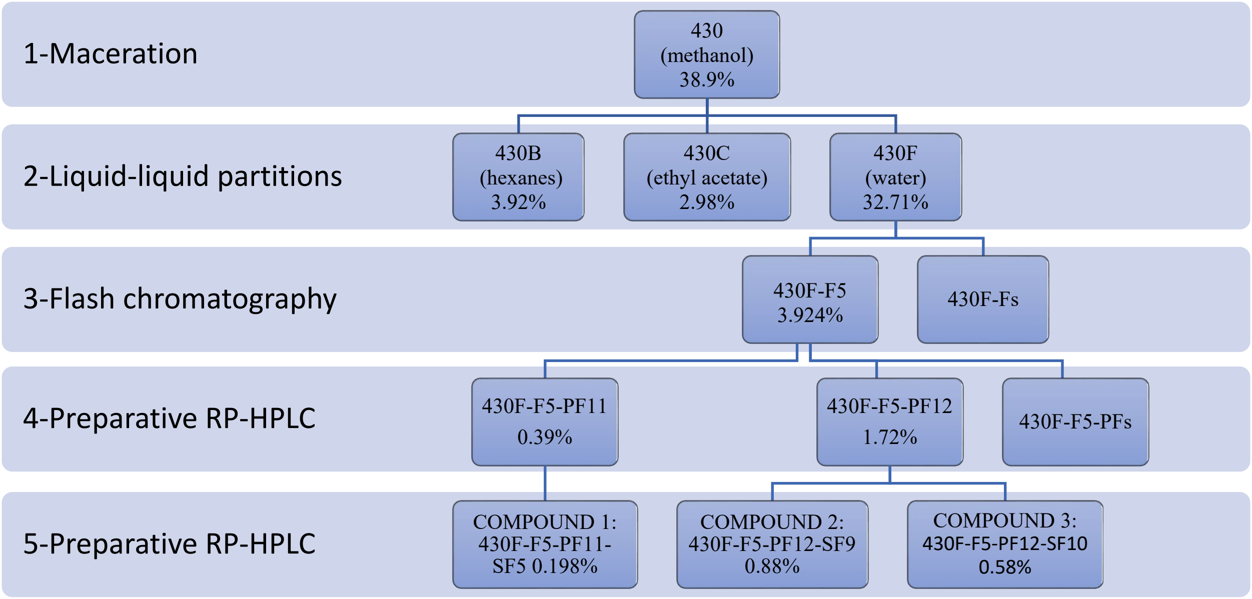

Figure S1. Fractionation strategy, with percent yields of the active fractions and compounds.

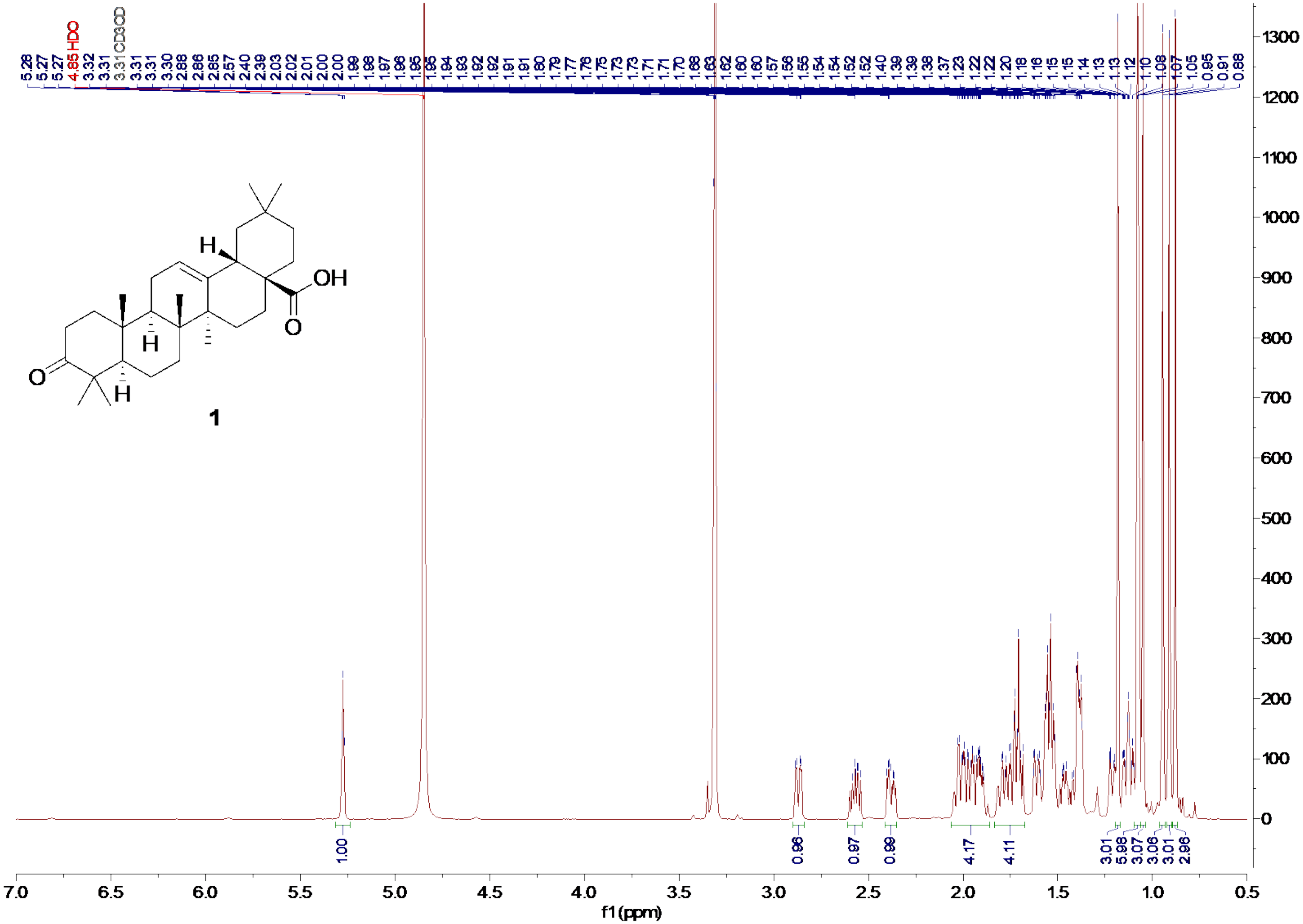

Figure S2. ^{1}H NMR (600 MHz, CD_3OD) spectrum of compound **1**.

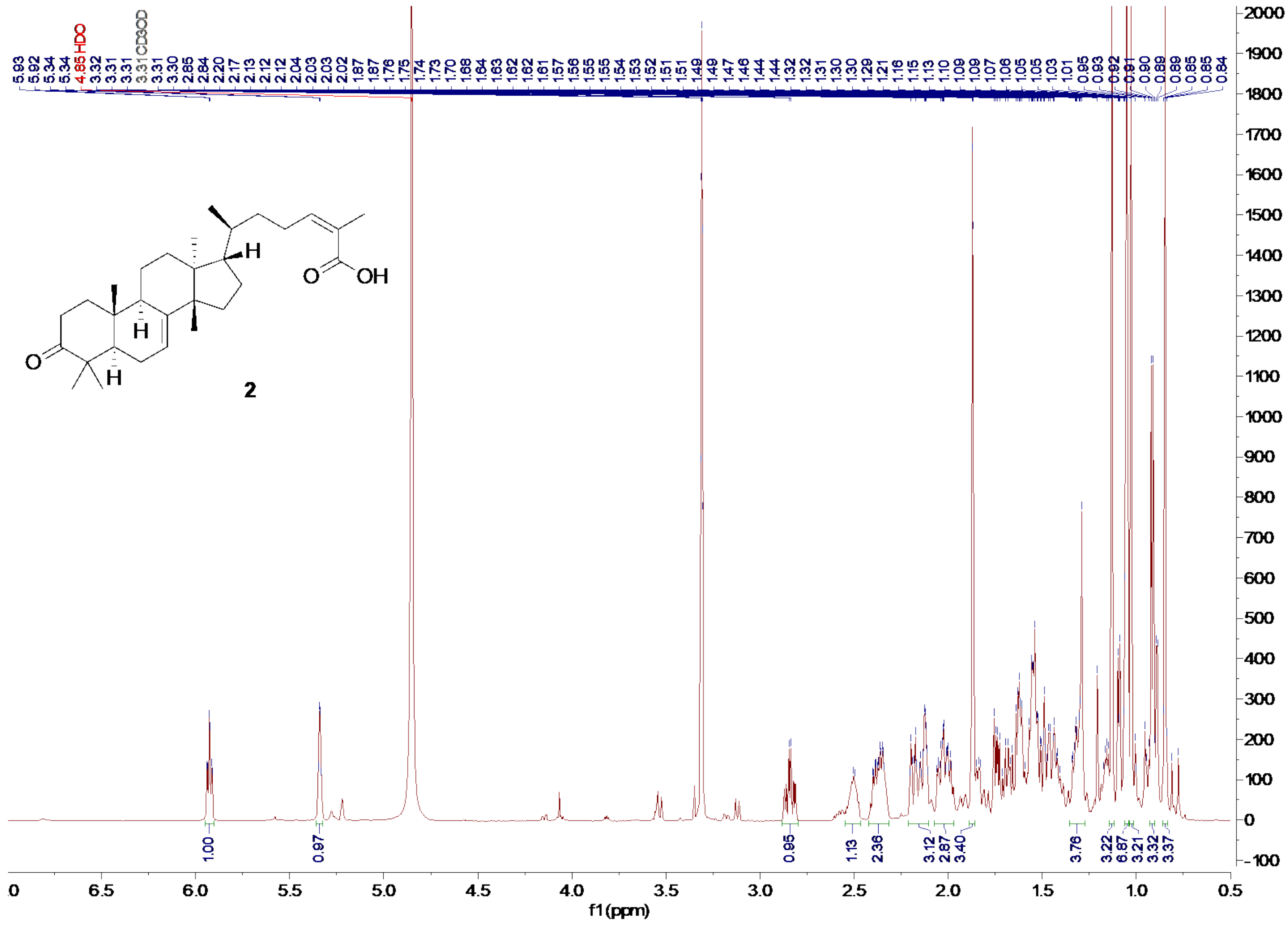

Figure S3. ^{1}H NMR (600 MHz, CD_3OD) spectrum of compound **2**.

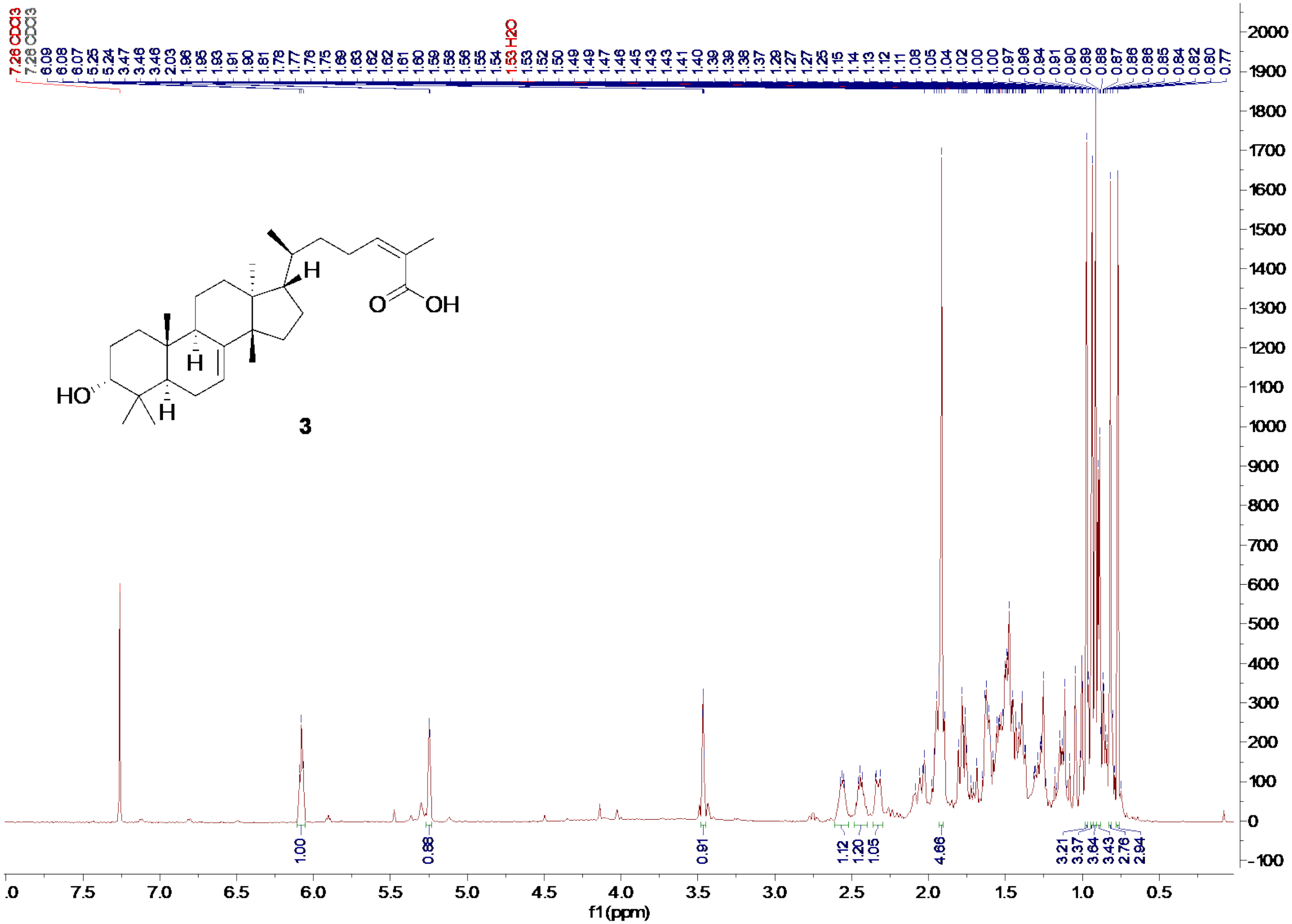

Figure S4. ^{1}H NMR (600 MHz, $CDCl_3$) spectrum of compound **3**.

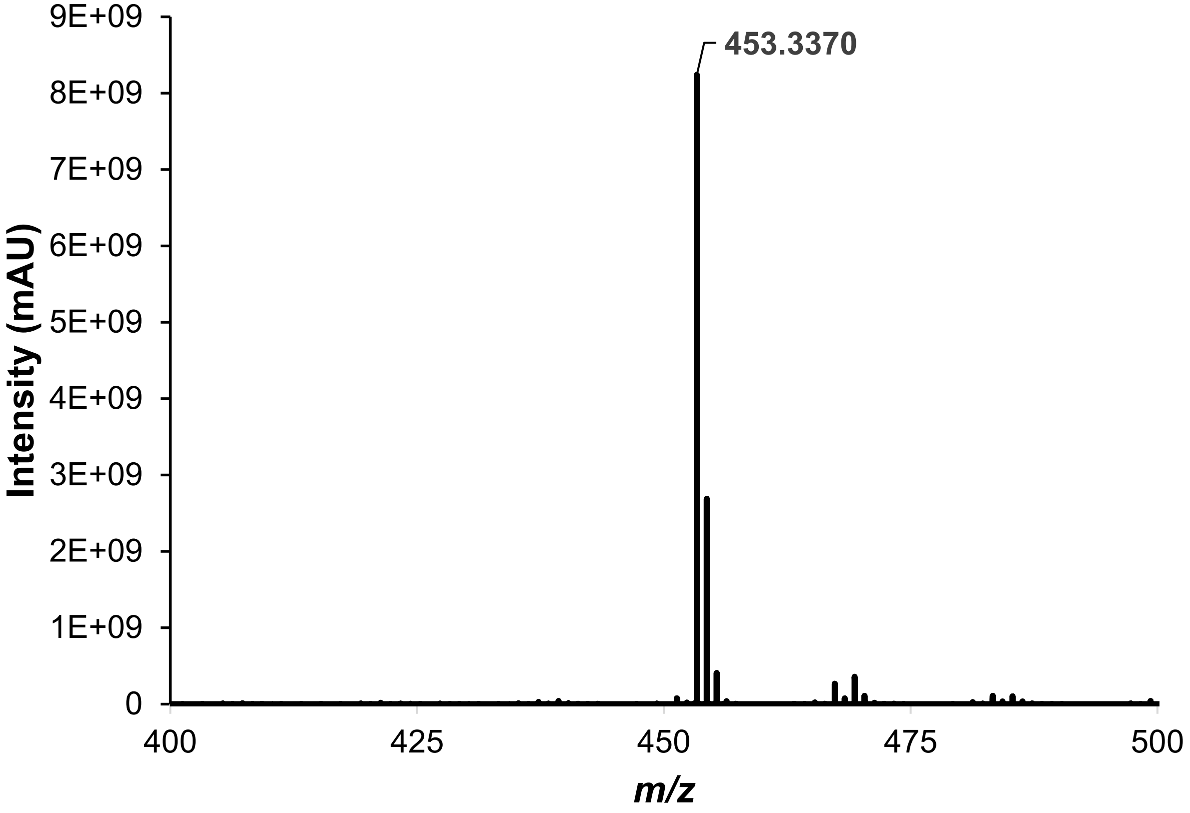

Peak Mass	Display Formula	RDB	Delta [ppm]	Theoretical mass	Combined Score	MS Cov. [%]
453.33696	$C_{30}H_{45}O_3$	8.5	-1.01	453.33742	96.53	99.31

Figure S5. HRMS-APCI negative mode spectra and empirical formula calculations for compound **1**.

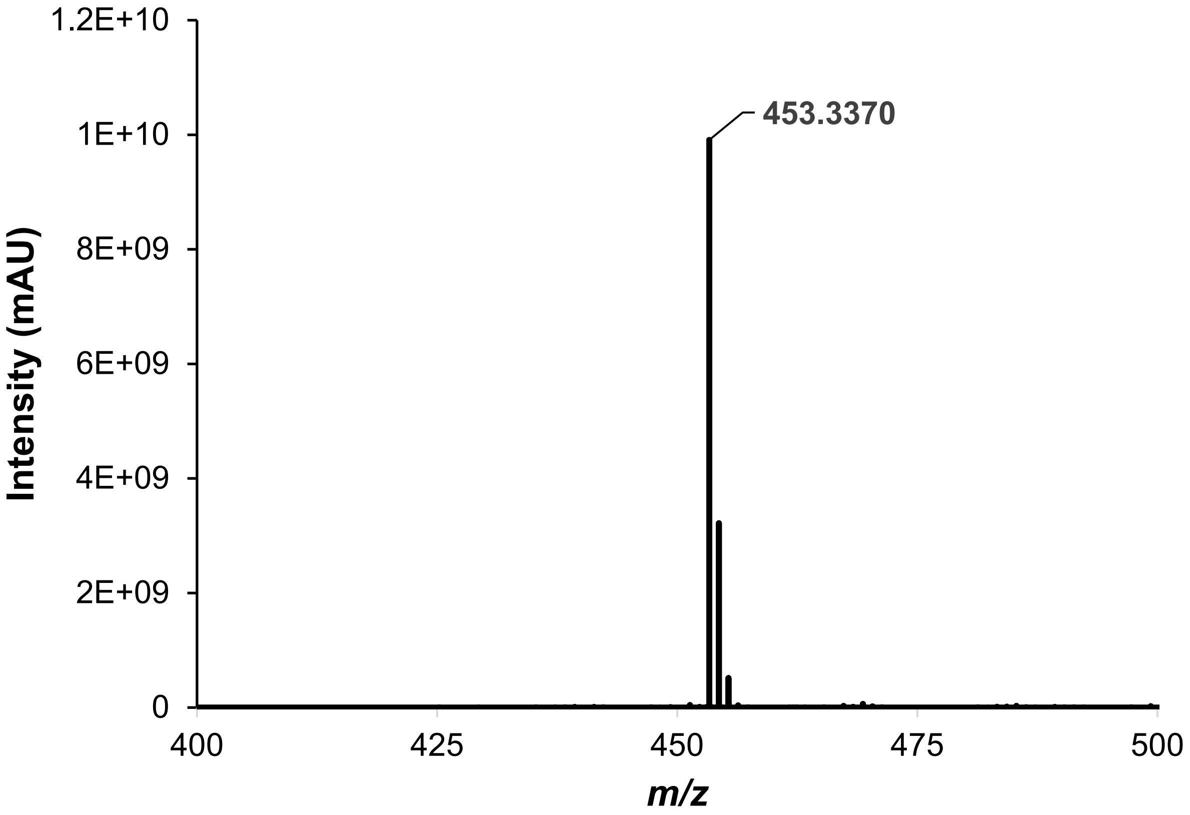

Peak Mass	Display Formula	RDB	Delta [ppm]	Theoretical. mass	Combined Score	MS Cov. [%]
453.33687	$C_{30}H_{45}O_3$	8.5	-1.2	453.33742	96.43	99.24

Figure S6. HRMS-APCI negative mode spectra and empirical formula calculations for compound **2**.

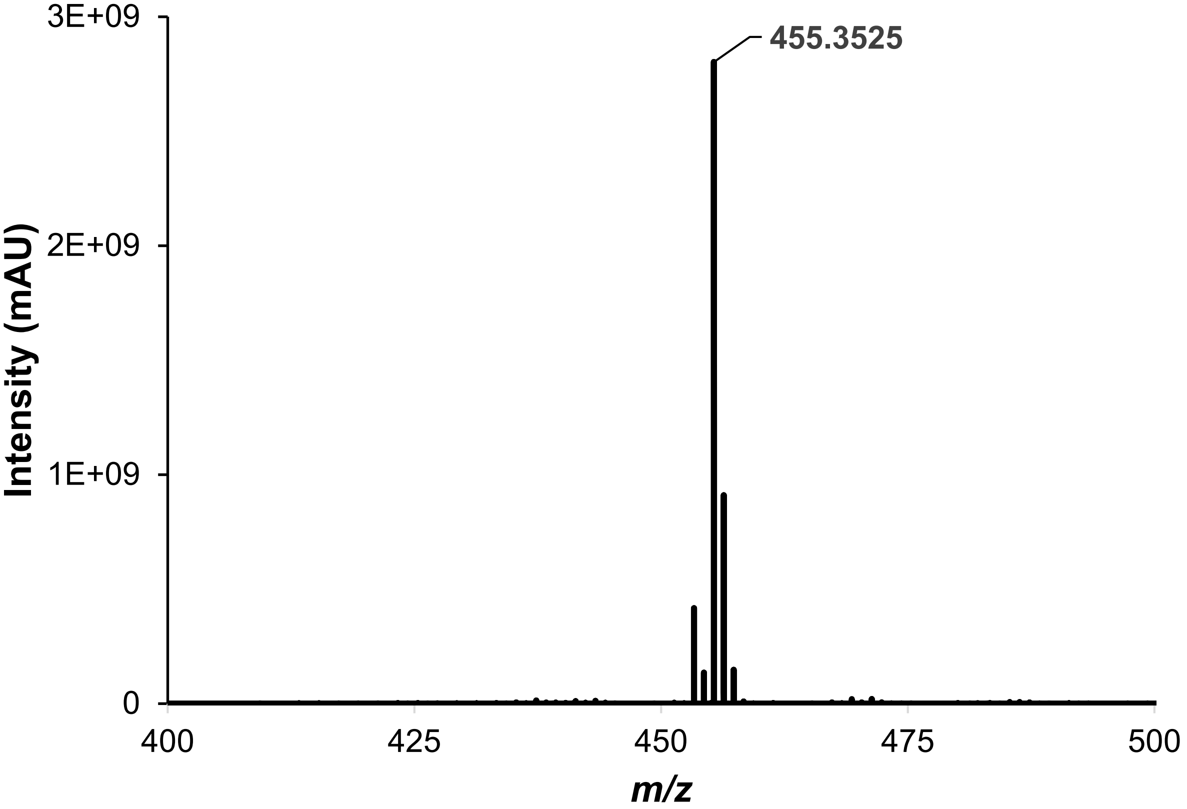

Peak Mass	Display Formula	RDB	Delta [ppm]	Theoretical. mass	Combined Score	MS Cov. [%]
455.35251	$C_{30}H_{47}O_3$	7.5	-1.22	455.35307	95.93	98.79

Figure S7. HRMS-APCI negative mode spectra and empirical formula calculations for compound **3**.

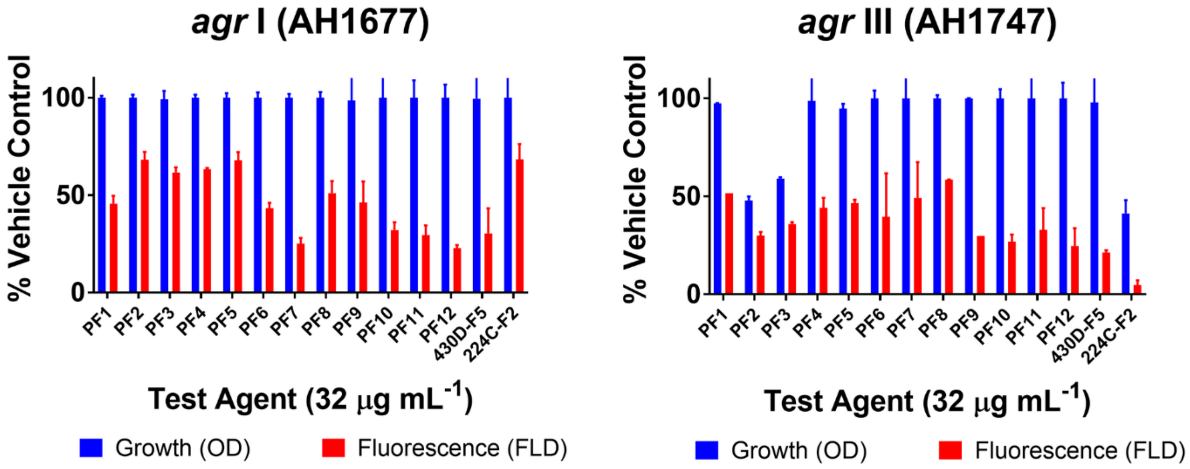

Figure S8. Quorum quenching activity step 4 (fractionation strategy, see **Figure S1**): fractions.

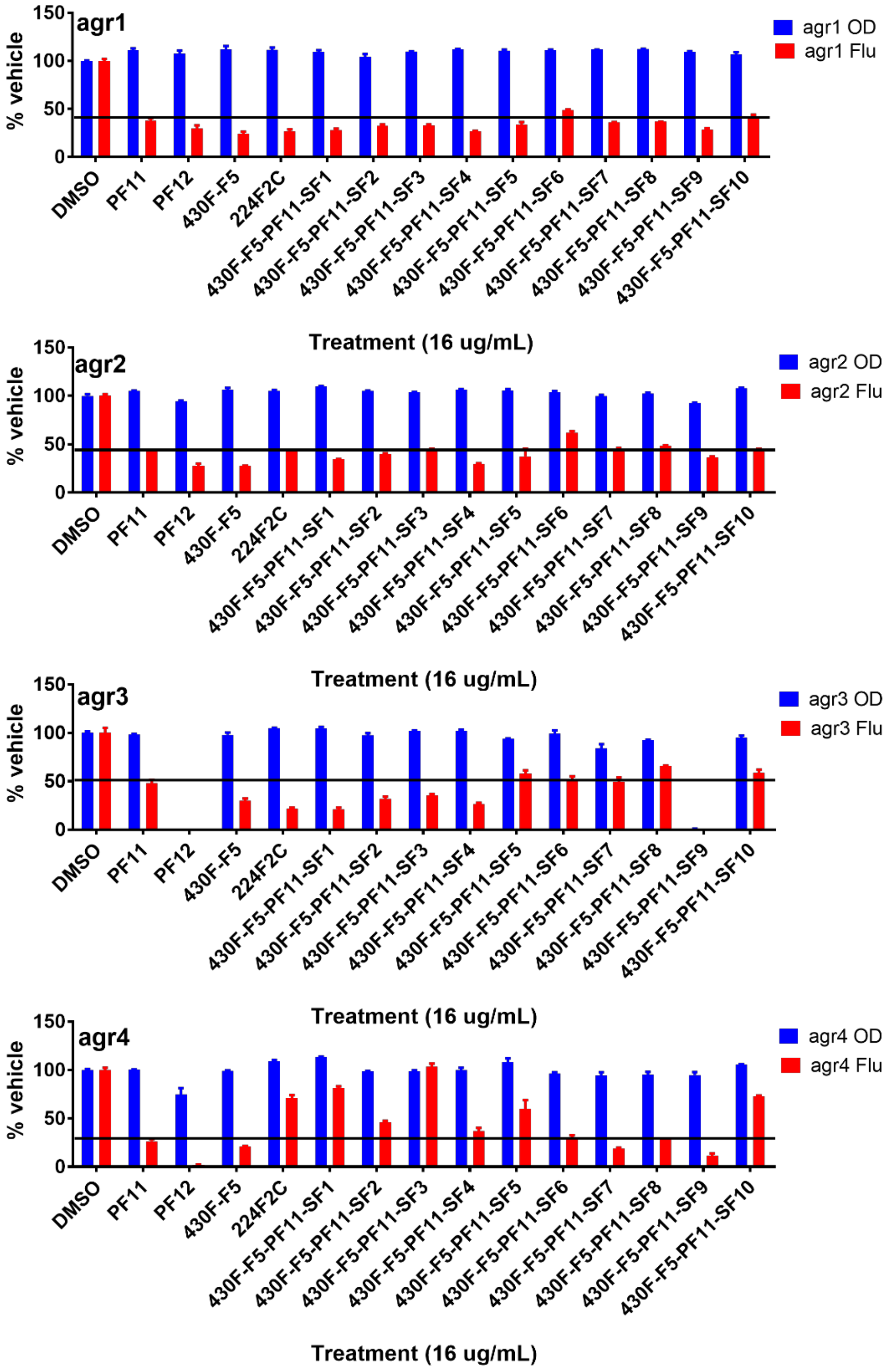

Figure S9. Quorum quenching activity step 5, 430F-F5-PF11 (fractionation strategy, see **Figure S1**): fractions and compounds.

*Note: Compound **1**: 430F-F5-PF11-SF5. 224F2c was used as a control.

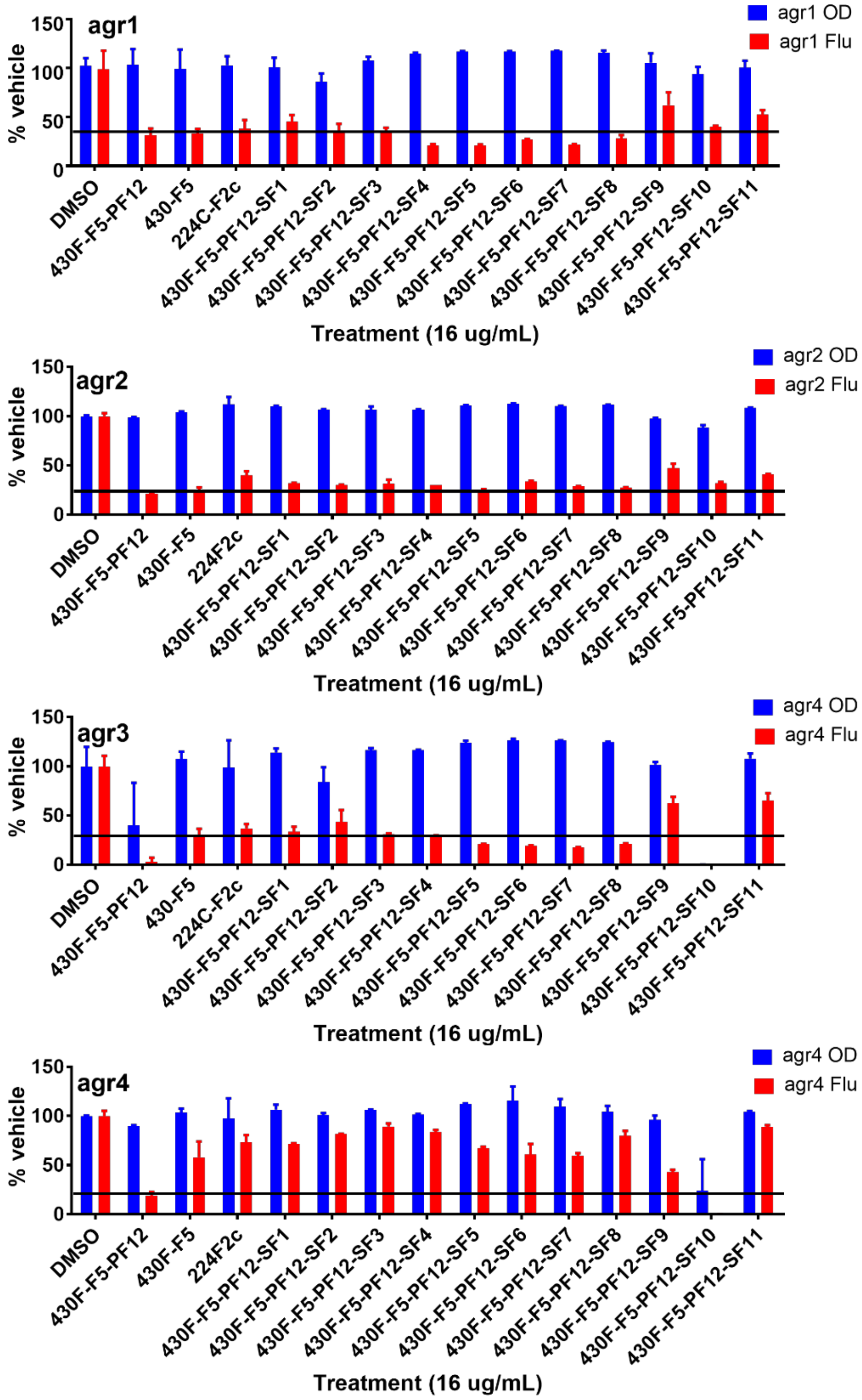

Figure S10. Quorum quenching activity step 5, 430F-F5-PF12 (fractionation strategy, see **Figure S1**): fractions and compounds.

*Note: Compound **2**: 430F-F5-PF12-SF9; Compound **3**: 430F-F5-PF12-SF10. 224F2c was used as a control.

Strain ID	Other Characteristics
AH430	SA502a + pDB59 Cm^R, yfp reporter, *agr* type II
AH1263 (LAC)	CA-MRSA, PFT USA300, *agr* type I
AH1677	AH845 + pDB59 Cm^R, yfp reporter, *agr* type I
AH1747	MW2 + pDB59 Cm^R, yfp reporter, *agr* type III
AH1872	MN EV(407) + pDB59 Cm^R, yfp reporter, *agr* type IV
NRS249	sea+, (lukE-lukD)+, hlgv+, associated with native valve endocarditis; SCCmec type IV, *agr* type I
UAMS-1	MSSA, osteomyelitis isolate, strong biofilm producer
UAMS-929	Isogenic *sarA* mutant of UAMS-1, biofilm deficient control strain
AH5116	Plasmid: pHC147. Genotype: pCM29-P*lukA*, Cm^R. Reference: Crosby *et al*. (2019)
AH5382	Plasmid: pHC178. Genotype: pCM29-P*gehB*, Cm^R. Reference: Crosby *et al*. (2019)
AH3613	Plasmid: pHC68. Genotype: *mgrA* P2-sGFP fusion, Erm^R. Reference: Crosby *et al*. (2016)
AH5101	Plasmid: pCM38. Genotype: P*nuc-sGFP,* Cm^R. Reference: Yan *et al*. (2019)
AH5095	Plasmid: pHC144. Genotype: pCM29-P*esxA*, Cm^R. Reference: Crosby *et al*. (2019)

Table S1. Profile of *Staphylococcus aureus* strains used in this study.

References:

Crosby, H.A., Schlievert, P.M., Merriman, J.A., King, J.M., Salgado-Pabon, W. and Horswill, A.R. (2016). The *Staphylococcus aureus* global regulator MgrA modulates clumping and virulence by controlling surface protein expression. *PLOS Pathogens*, 12, e1005604.

Crosby, H.A., Tiwari, N., Kwiecinski, J.M., Xu, Z., Dykstra, A., Jenul, C., Fuentes, E.J., and Horswill, A.R. (2019). The *Staphylococcus aureus* ArlRS two-component system regulates virulence factor expression through MgrA. *Molecular Microbiology*. Available at: doi:10.111/mmi.14404

Yan, H., Wang, Q., Teng, M. and Li, X. (2019) The DNA- binding mechanism of the TCS response regulator ArlR from *Staphylococcus aureus*. *Journal of Structural Biology*. Available at: doi:10.1016/j.jsb.2019.09.005.

checkCIF/PLATON report

Structure factors have been supplied for datablock(s) pf11-sf5

THIS REPORT IS FOR GUIDANCE ONLY. IF USED AS PART OF A REVIEW PROCEDURE FOR PUBLICATION, IT SHOULD NOT REPLACE THE EXPERTISE OF AN EXPERIENCED CRYSTALLOGRAPHIC REFEREE.

No syntax errors found. CIF dictionary Interpreting this report

Datablock: pf11-sf5

```
Bond precision:  C-C = 0.0037 A          Wavelength=1.54184

Cell:           a=11.8169(3)    b=7.3667(2)       c=34.0413(12)
                alpha=90        beta=95.459(3)    gamma=90
Temperature:    106 K

                Calculated                 Reported
Volume          2949.91(15)                2949.88(16)
Space group     I 2                        I 1 2 1
Hall group      I 2y                       I 2y
Moiety formula  C30 H46 O3, 2(C H4 O)      2(C H4 O), C30 H46 O3
Sum formula     C32 H54 O5                 C32 H54 O5
Mr              518.75                     518.75
Dx,g cm-3       1.168                      1.168
Z               4                          4
Mu (mm-1)       0.601                      0.601
F000            1144.0                     1144.0
F000'           1147.18
h,k,lmax        14,8,40                    14,8,40
Nref            5400[ 2928]                5239
Tmin,Tmax       0.918,0.952                0.659,1.000
Tmin'           0.817

Correction method= # Reported T Limits: Tmin=0.659 Tmax=1.000
AbsCorr = GAUSSIAN

Data completeness= 1.79/0.97      Theta(max)= 68.217

R(reflections)= 0.0410( 4942)     wR2(reflections)= 0.1133( 5239)

S = 1.057                  Npar= 356
```

The following ALERTS were generated. Each ALERT has the format
test-name_ALERT_alert-type_alert-level.
Click on the hyperlinks for more details of the test.

Alert level C

```
PLAT412_ALERT_2_C Short Intra XH3 .. XHn     H3A      ..H23A     .        1.87 Ang.
                                                      x,y,z  =      1_555 Check
PLAT911_ALERT_3_C Missing FCF Refl Between Thmin & STh/L=    0.600          21 Report
```

Alert level G

```
PLAT002_ALERT_2_G Number of Distance or Angle Restraints on AtSite          6 Note
PLAT042_ALERT_1_G Calc. and Reported MoietyFormula Strings  Differ     Please Check
PLAT172_ALERT_4_G The CIF-Embedded .res File Contains DFIX Records          3 Report
PLAT720_ALERT_4_G Number of Unusual/Non-Standard Labels ..........          6 Note
PLAT791_ALERT_4_G Model has Chirality at C1          (Sohnke SpGr)          R Verify
PLAT791_ALERT_4_G Model has Chirality at C2          (Sohnke SpGr)          S Verify
PLAT791_ALERT_4_G Model has Chirality at C5          (Sohnke SpGr)          S Verify
PLAT791_ALERT_4_G Model has Chirality at C6          (Sohnke SpGr)          S Verify
PLAT791_ALERT_4_G Model has Chirality at C11         (Sohnke SpGr)          R Verify
PLAT791_ALERT_4_G Model has Chirality at C12         (Sohnke SpGr)          R Verify
PLAT791_ALERT_4_G Model has Chirality at C13         (Sohnke SpGr)          R Verify
PLAT860_ALERT_3_G Number of Least-Squares Restraints .............          4 Note
PLAT912_ALERT_4_G Missing # of FCF Reflections Above STh/L=  0.600          9 Note
PLAT933_ALERT_2_G Number of OMIT Records in Embedded .res File ...          7 Note
PLAT941_ALERT_3_G Average HKL Measurement Multiplicity ...........        4.9 Low
PLAT978_ALERT_2_G Number C-C Bonds with Positive Residual Density.          8 Info
```

```
   0 ALERT level A = Most likely a serious problem - resolve or explain
   0 ALERT level B = A potentially serious problem, consider carefully
   2 ALERT level C = Check. Ensure it is not caused by an omission or oversight
  16 ALERT level G = General information/check it is not something unexpected

   1 ALERT type 1 CIF construction/syntax error, inconsistent or missing data
   4 ALERT type 2 Indicator that the structure model may be wrong or deficient
   3 ALERT type 3 Indicator that the structure quality may be low
  10 ALERT type 4 Improvement, methodology, query or suggestion
   0 ALERT type 5 Informative message, check
```

It is advisable to attempt to resolve as many as possible of the alerts in all categories. Often the minor alerts point to easily fixed oversights, errors and omissions in your CIF or refinement strategy, so attention to these fine details can be worthwhile. In order to resolve some of the more serious problems it may be necessary to carry out additional measurements or structure refinements. However, the purpose of your study may justify the reported deviations and the more serious of these should normally be commented upon in the discussion or experimental section of a paper or in the "special_details" fields of the CIF. checkCIF was carefully designed to identify outliers and unusual parameters, but every test has its limitations and alerts that are not important in a particular case may appear. Conversely, the absence of alerts does not guarantee there are no aspects of the results needing attention. It is up to the individual to critically assess their own results and, if necessary, seek expert advice.

Publication of your CIF in IUCr journals

A basic structural check has been run on your CIF. These basic checks will be run on all CIFs submitted for publication in IUCr journals (*Acta Crystallographica*, *Journal of Applied Crystallography*, *Journal of Synchrotron Radiation*); however, if you intend to submit to *Acta Crystallographica Section C* or *E* or *IUCrData*, you should make sure that full publication checks are run on the final version of your CIF prior to submission.

Publication of your CIF in other journals

Please refer to the *Notes for Authors* of the relevant journal for any special instructions relating to CIF submission.

PLATON version of 16/04/2020; check.def file version of 09/03/2020

Datablock pf11-sf5 - ellipsoid plot

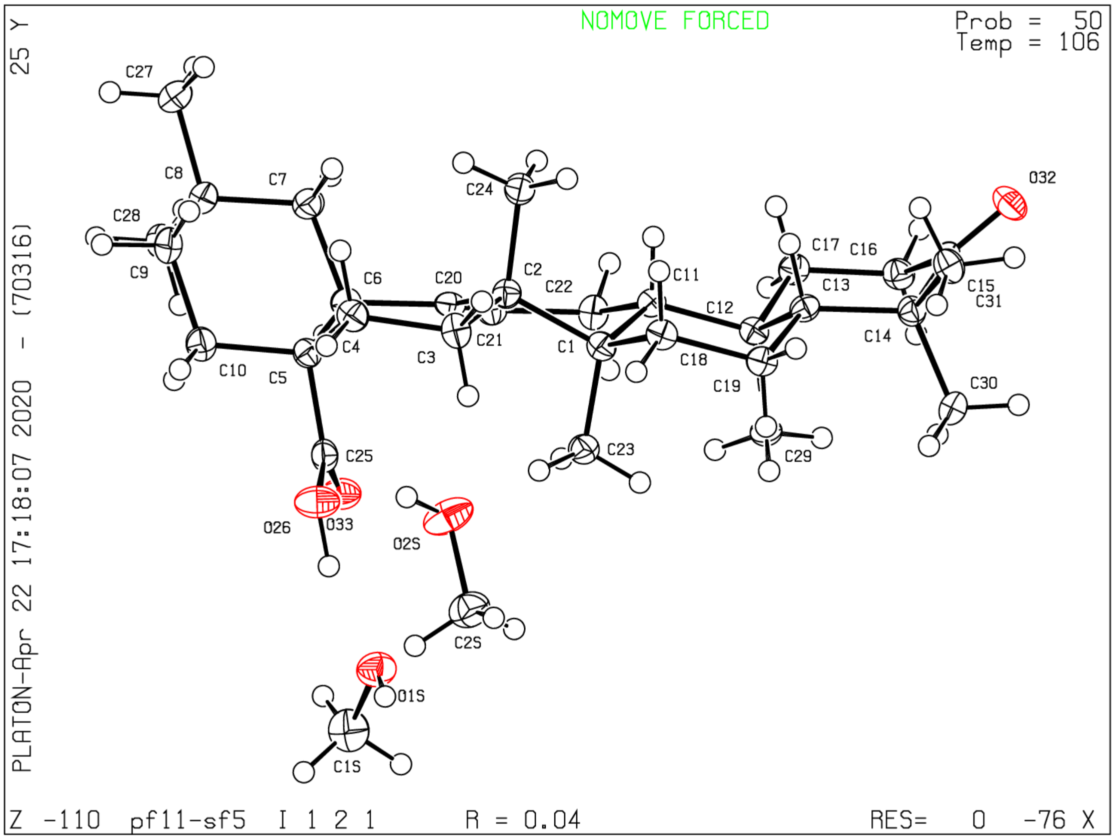

checkCIF/PLATON report

Structure factors have been supplied for datablock(s) pf12-sf9

THIS REPORT IS FOR GUIDANCE ONLY. IF USED AS PART OF A REVIEW PROCEDURE FOR PUBLICATION, IT SHOULD NOT REPLACE THE EXPERTISE OF AN EXPERIENCED CRYSTALLOGRAPHIC REFEREE.

No syntax errors found. CIF dictionary Interpreting this report

Datablock: pf12-sf9

```
Bond precision:  C-C = 0.0029 A          Wavelength=1.54184

Cell:           a=6.81969(10)    b=19.7846(3)        c=19.4400(3)
                alpha=90         beta=94.4735(13)    gamma=90
Temperature:    107 K

                Calculated                  Reported
Volume          2614.95(7)                  2614.95(7)
Space group     P 21                        P 1 21 1
Hall group      P 2yb                       P 2yb
Moiety formula  C30 H46 O3                  4(C30 H46 O3)
Sum formula     C30 H46 O3                  C120 H184 O12
Mr              454.67                      1818.66
Dx,g cm-3       1.155                       1.155
Z               4                           1
Mu (mm-1)       0.557                       0.557
F000            1000.0                      1000.0
F000'           1002.65
h,k,lmax        8,24,24                     8,24,24
Nref            11027[ 5679]                10230
Tmin,Tmax       0.902,0.933                 0.491,1.000
Tmin'           0.762

Correction method= # Reported T Limits: Tmin=0.491 Tmax=1.000
AbsCorr = GAUSSIAN

Data completeness= 1.80/0.93      Theta(max)= 76.669

R(reflections)= 0.0349( 9980)     wR2(reflections)= 0.0938( 10230)

S = 1.022                   Npar= 610
```

The following ALERTS were generated. Each ALERT has the format
test-name_ALERT_alert-type_alert-level.
Click on the hyperlinks for more details of the test.

Alert level C

```
PLAT911_ALERT_3_C Missing FCF Refl Between Thmin & STh/L=    0.600          7 Report
PLAT915_ALERT_3_C No Flack x Check Done: Low Friedel Pair Coverage         88 %
```

Alert level G

```
PLAT007_ALERT_5_G Number of Unrefined Donor-H Atoms ..............          2 Report
PLAT042_ALERT_1_G Calc. and Reported MoietyFormula Strings  Differ     Please Check
PLAT045_ALERT_1_G Calculated and Reported Z Differ by a Factor ...       4.00 Check
PLAT380_ALERT_4_G Incorrectly? Oriented X(sp2)-Methyl Moiety .....       C25B Check
PLAT720_ALERT_4_G Number of Unusual/Non-Standard Labels ..........          9 Note
PLAT791_ALERT_4_G Model has Chirality at C1          (Sohnke SpGr)          S Verify
PLAT791_ALERT_4_G Model has Chirality at C1B         (Sohnke SpGr)          S Verify
PLAT791_ALERT_4_G Model has Chirality at C2          (Sohnke SpGr)          S Verify
PLAT791_ALERT_4_G Model has Chirality at C2B         (Sohnke SpGr)          S Verify
PLAT791_ALERT_4_G Model has Chirality at C5          (Sohnke SpGr)          S Verify
PLAT791_ALERT_4_G Model has Chirality at C5B         (Sohnke SpGr)          S Verify
PLAT791_ALERT_4_G Model has Chirality at C8          (Sohnke SpGr)          S Verify
PLAT791_ALERT_4_G Model has Chirality at C8B         (Sohnke SpGr)          S Verify
PLAT791_ALERT_4_G Model has Chirality at C9          (Sohnke SpGr)          R Verify
PLAT791_ALERT_4_G Model has Chirality at C9B         (Sohnke SpGr)          R Verify
PLAT791_ALERT_4_G Model has Chirality at C10         (Sohnke SpGr)          R Verify
PLAT791_ALERT_4_G Model has Chirality at C10B        (Sohnke SpGr)          R Verify
PLAT791_ALERT_4_G Model has Chirality at C20         (Sohnke SpGr)          S Verify
PLAT791_ALERT_4_G Model has Chirality at C20B        (Sohnke SpGr)          S Verify
PLAT910_ALERT_3_G Missing # of FCF Reflection(s) Below Theta(Min).          1 Note
PLAT912_ALERT_4_G Missing # of FCF Reflections Above STh/L=  0.600        171 Note
PLAT933_ALERT_2_G Number of OMIT Records in Embedded .res File ...         11 Note
PLAT978_ALERT_2_G Number C-C Bonds with Positive Residual Density.         21 Info
```

```
   0 ALERT level A = Most likely a serious problem - resolve or explain
   0 ALERT level B = A potentially serious problem, consider carefully
   2 ALERT level C = Check. Ensure it is not caused by an omission or oversight
  23 ALERT level G = General information/check it is not something unexpected

   2 ALERT type 1 CIF construction/syntax error, inconsistent or missing data
   2 ALERT type 2 Indicator that the structure model may be wrong or deficient
   3 ALERT type 3 Indicator that the structure quality may be low
  17 ALERT type 4 Improvement, methodology, query or suggestion
   1 ALERT type 5 Informative message, check
```

It is advisable to attempt to resolve as many as possible of the alerts in all categories. Often the minor alerts point to easily fixed oversights, errors and omissions in your CIF or refinement strategy, so attention to these fine details can be worthwhile. In order to resolve some of the more serious problems it may be necessary to carry out additional measurements or structure refinements. However, the purpose of your study may justify the reported deviations and the more serious of these should normally be commented upon in the discussion or experimental section of a paper or in the "special_details" fields of the CIF. checkCIF was carefully designed to identify outliers and unusual parameters, but every test has its limitations and alerts that are not important in a particular case may appear. Conversely, the absence of alerts does not guarantee there are no aspects of the results needing attention. It is up to the individual to critically assess their own results and, if necessary, seek expert advice.

Publication of your CIF in IUCr journals

A basic structural check has been run on your CIF. These basic checks will be run on all CIFs submitted for publication in IUCr journals (*Acta Crystallographica*, *Journal of Applied Crystallography*, *Journal of Synchrotron Radiation*); however, if you intend to submit to *Acta Crystallographica Section C* or *E* or *IUCrData*, you should make sure that full publication checks are run on the final version of your CIF prior to submission.

Publication of your CIF in other journals

Please refer to the *Notes for Authors* of the relevant journal for any special instructions relating to CIF submission.

PLATON version of 16/04/2020; check.def file version of 09/03/2020

Datablock pf12-sf9 - ellipsoid plot

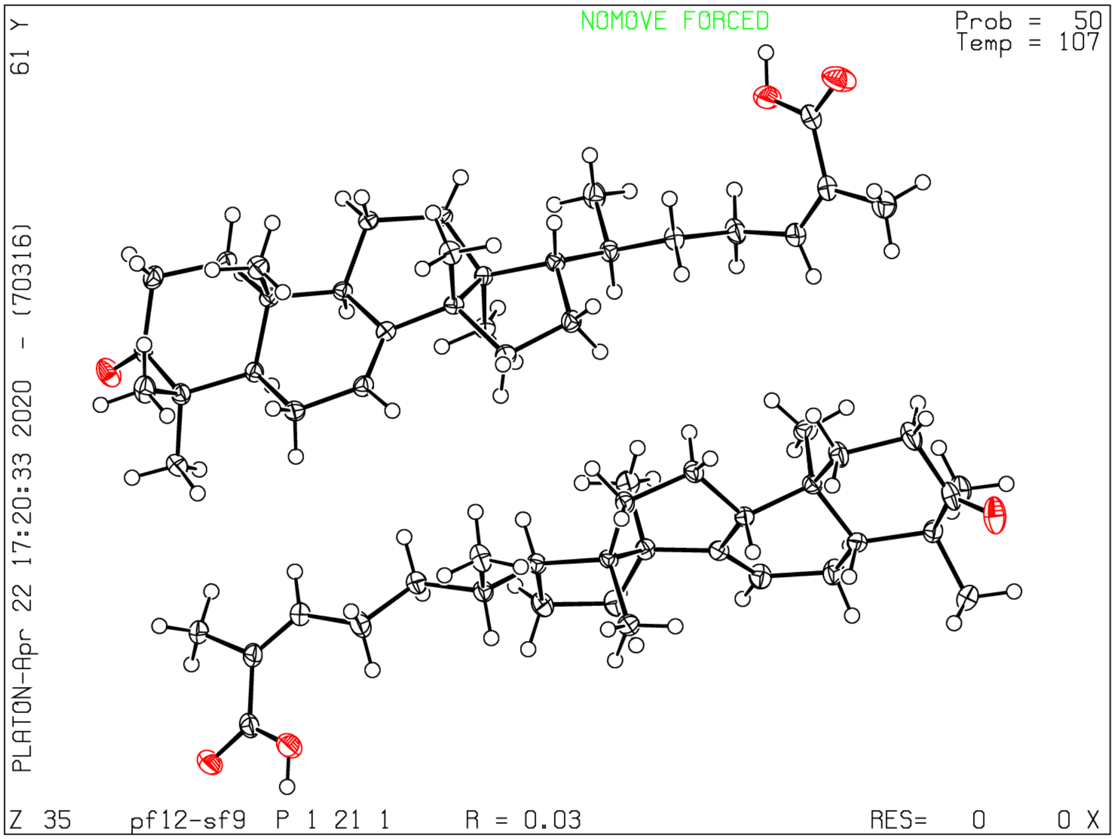

checkCIF/PLATON report

Structure factors have been supplied for datablock(s) pf11-sf10

THIS REPORT IS FOR GUIDANCE ONLY. IF USED AS PART OF A REVIEW PROCEDURE FOR PUBLICATION, IT SHOULD NOT REPLACE THE EXPERTISE OF AN EXPERIENCED CRYSTALLOGRAPHIC REFEREE.

No syntax errors found.   CIF dictionary   Interpreting this report

Datablock: pf11-sf10

```
Bond precision:  C-C = 0.0052 A          Wavelength=1.54184

Cell:            a=7.43165(12)     b=12.8741(2)     c=59.8281(12)
                 alpha=90          beta=90          gamma=90
Temperature:     102 K

                Calculated                 Reported
Volume          5724.10(17)                5724.12(18)
Space group     P 21 21 21                 P 21 21 21
Hall group      P 2ac 2ab                  P 2ac 2ab
Moiety formula  C30 H48 O3, C H4 O         C30 H48 O3, C H4 O
Sum formula     C31 H52 O4                 C31 H52 O4
Mr              488.73                     488.72
Dx,g cm-3       1.134                      1.134
Z               8                          8
Mu (mm-1)       0.564                      0.564
F000            2160.0                     2160.0
F000'           2165.83
h,k,lmax        8,15,72                    8,15,71
Nref            10450[ 5944]               10360
Tmin,Tmax       0.804,0.978                0.543,1.000
Tmin'           0.746

Correction method= # Reported T Limits: Tmin=0.543 Tmax=1.000
AbsCorr = GAUSSIAN

Data completeness= 1.74/0.99      Theta(max)= 68.243

R(reflections)= 0.0503( 8100)     wR2(reflections)= 0.1301( 10360)

S = 1.050                   Npar= 654
```

The following ALERTS were generated. Each ALERT has the format
test-name_ALERT_alert-type_alert-level.
Click on the hyperlinks for more details of the test.

Alert level C

```
PLAT031_ALERT_4_C Refined Extinction Parameter Within Range ......      3.100 Sigma
PLAT340_ALERT_3_C Low Bond Precision on  C-C Bonds ...............    0.00518 Ang.
PLAT417_ALERT_2_C Short Inter D-H..H-D       H28      ..H35      .       2.12 Ang.
                                                x,y,z  =      1_555 Check
PLAT911_ALERT_3_C Missing FCF Refl Between Thmin & STh/L=    0.600         14 Report
```

Alert level G

```
PLAT007_ALERT_5_G Number of Unrefined Donor-H Atoms ..............          6 Report
PLAT720_ALERT_4_G Number of Unusual/Non-Standard Labels ..........          8 Note
PLAT790_ALERT_4_G Centre of Gravity not Within Unit Cell: Resd.  #          2 Note
              C30 H48 O3
PLAT790_ALERT_4_G Centre of Gravity not Within Unit Cell: Resd.  #          4 Note
              C H4 O
PLAT791_ALERT_4_G Model has Chirality at C1          (Sohnke SpGr)          S Verify
PLAT791_ALERT_4_G Model has Chirality at C1B         (Sohnke SpGr)          S Verify
PLAT791_ALERT_4_G Model has Chirality at C2          (Sohnke SpGr)          S Verify
PLAT791_ALERT_4_G Model has Chirality at C2B         (Sohnke SpGr)          S Verify
PLAT791_ALERT_4_G Model has Chirality at C5          (Sohnke SpGr)          R Verify
PLAT791_ALERT_4_G Model has Chirality at C5B         (Sohnke SpGr)          R Verify
PLAT791_ALERT_4_G Model has Chirality at C6          (Sohnke SpGr)          R Verify
PLAT791_ALERT_4_G Model has Chirality at C6B         (Sohnke SpGr)          R Verify
PLAT791_ALERT_4_G Model has Chirality at C9          (Sohnke SpGr)          R Verify
PLAT791_ALERT_4_G Model has Chirality at C9B         (Sohnke SpGr)          R Verify
PLAT791_ALERT_4_G Model has Chirality at C11         (Sohnke SpGr)          R Verify
PLAT791_ALERT_4_G Model has Chirality at C11B        (Sohnke SpGr)          R Verify
PLAT791_ALERT_4_G Model has Chirality at C15         (Sohnke SpGr)          S Verify
PLAT791_ALERT_4_G Model has Chirality at C15B        (Sohnke SpGr)          S Verify
PLAT791_ALERT_4_G Model has Chirality at C18         (Sohnke SpGr)          S Verify
PLAT791_ALERT_4_G Model has Chirality at C18B        (Sohnke SpGr)          S Verify
PLAT850_ALERT_4_G Check Flack Parameter Exact Value 0.00 and s.u.        0.14 Check
PLAT910_ALERT_3_G Missing # of FCF Reflection(s) Below Theta(Min).          2 Note
PLAT912_ALERT_4_G Missing # of FCF Reflections Above STh/L=  0.600          6 Note
PLAT933_ALERT_2_G Number of OMIT Records in Embedded .res File ...          2 Note
PLAT978_ALERT_2_G Number C-C Bonds with Positive Residual Density.          3 Info
```

```
   0 ALERT level A = Most likely a serious problem - resolve or explain
   0 ALERT level B = A potentially serious problem, consider carefully
   4 ALERT level C = Check. Ensure it is not caused by an omission or oversight
  25 ALERT level G = General information/check it is not something unexpected

   0 ALERT type 1 CIF construction/syntax error, inconsistent or missing data
   3 ALERT type 2 Indicator that the structure model may be wrong or deficient
   3 ALERT type 3 Indicator that the structure quality may be low
  22 ALERT type 4 Improvement, methodology, query or suggestion
   1 ALERT type 5 Informative message, check
```

It is advisable to attempt to resolve as many as possible of the alerts in all categories. Often the minor alerts point to easily fixed oversights, errors and omissions in your CIF or refinement strategy, so attention to these fine details can be worthwhile. In order to resolve some of the more serious problems it may be necessary to carry out additional measurements or structure refinements. However, the purpose of your study may justify the reported deviations and the more serious of these should normally be commented upon in the discussion or experimental section of a paper or in the "special_details" fields of the CIF. checkCIF was carefully designed to identify outliers and unusual parameters, but every test has its limitations and alerts that are not important in a particular case may appear. Conversely, the absence of alerts does not guarantee there are no aspects of the results needing attention. It is up to the individual to critically assess their own results and, if necessary, seek expert advice.

Publication of your CIF in IUCr journals

A basic structural check has been run on your CIF. These basic checks will be run on all CIFs submitted for publication in IUCr journals (*Acta Crystallographica*, *Journal of Applied Crystallography*, *Journal of Synchrotron Radiation*); however, if you intend to submit to *Acta Crystallographica Section C* or *E* or *IUCrData*, you should make sure that full publication checks are run on the final version of your CIF prior to submission.

Publication of your CIF in other journals

Please refer to the *Notes for Authors* of the relevant journal for any special instructions relating to CIF submission.

PLATON version of 16/04/2020; check.def file version of 09/03/2020

Datablock pf11-sf10 - ellipsoid plot

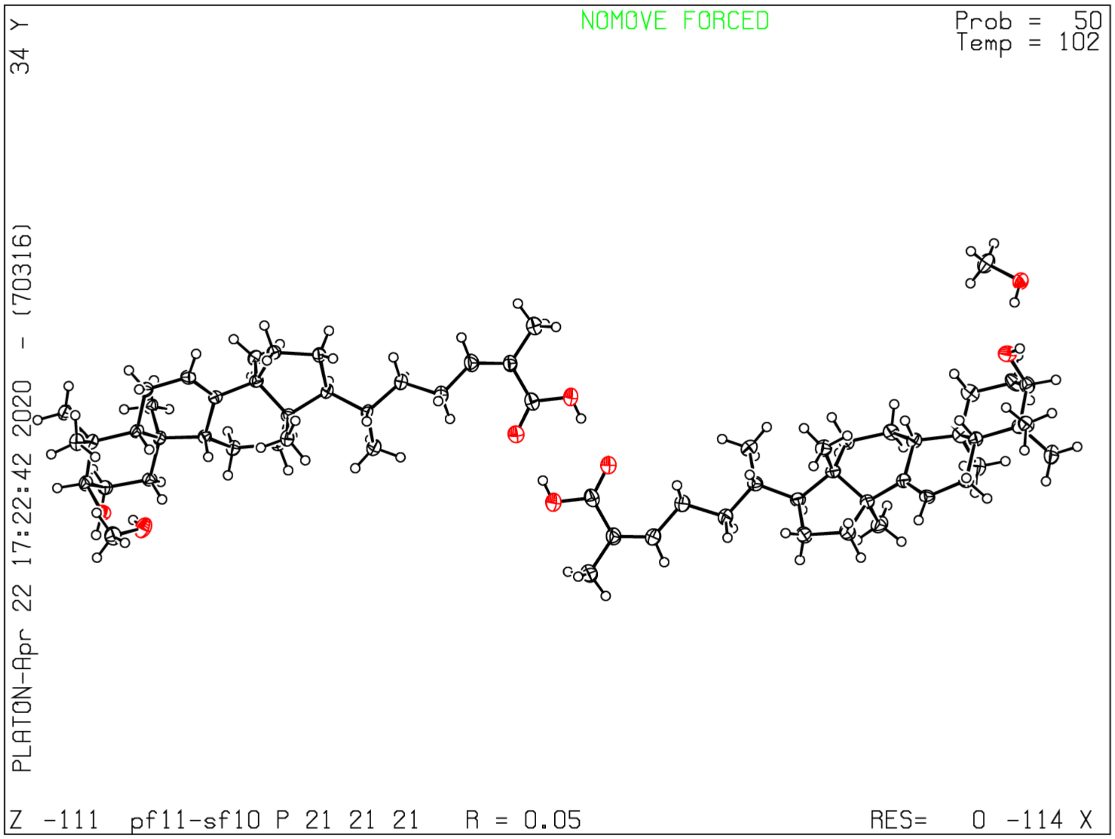

Supplement: Supplementary file 1 — Supplementary Information. [file 41598_2020_65080_MOESM1_ESM.pdf]
